# Video Intervention and Goals-of-Care Documentation in Hospitalized Older Adults: The VIDEO-PCE Randomized Clinical Trial

**DOI:** 10.1001/jamanetworkopen.2023.32556

**Published:** 2023-09-11

**Authors:** Angelo E. Volandes, Sophia N. Zupanc, Joshua R. Lakin, Howard J. Cabral, Edith A. Burns, Maria T. Carney, Santiago Lopez, Jennifer Itty, Kaitlin Emmert, Narda J. Martin, Therese Cole, Alexandra Dobie, Traci Cucinotta, Milton Joel, Lisa B. Caruso, Lori Henault, Julianne N. Dugas, Kristina Astone, Michael Winter, Na Wang, Aretha Delight Davis, Cynthia Garde, Perla Macip Rodriguez, Areej El-Jawahri, Edward T. Moseley, Sophiya Das, Kate Sciacca, Ana Maria Ramirez, Valeria Gromova, Sherene Lambert, Shreya Sanghani, Charlotta Lindvall, Michael K. Paasche-Orlow

**Affiliations:** 1 Harvard Medical School, Boston, Massachusetts; 2 Department of Medicine, Massachusetts General Hospital, Boston; 3 ACP Decisions, Waban, Massachusetts; 4 Department of Psychosocial Oncology and Palliative Care, Dana-Farber Cancer Institute, Boston, Massachusetts; 5 Department of Medicine, Brigham and Women’s Hospital, Boston, Massachusetts; 6 Department of Biostatistics, Boston University School of Public Health, Boston, Massachusetts; 7 Institute of Health System Science, Feinstein Institutes for Medical Research, Northwell Health, Manhasset, New York; 8 Department of Medicine, Donald and Barbara Zucker School of Medicine at Hofstra/Northwell, New Hyde Park, New York; 9 Section of General Internal Medicine, Boston Medical Center, Boston University Chobanian & Avedisian School of Medicine, Boston, Massachusetts; 10 Palliative Care, Boston Medical Center, Boston, Massachusetts; 11 Department of Medicine, Section of Geriatrics, Boston Medical Center, Boston University Chobanian & Avedisian School of Medicine, Boston, Massachusetts; 12 Biostatistics and Epidemiology Data Analytics Center, Boston University School of Public Health, Boston, Massachusetts; 13 Department of Medicine, Tufts University School of Medicine, Tufts Medical Center, Boston, Massachusetts

## Abstract

**Importance:**

Despite the benefits of goals-of-care (GOC) communication, many hospitalized individuals never communicate their goals or preferences to clinicians.

**Objective:**

To assess whether a GOC video intervention delivered by palliative care educators (PCEs) increased the rate of GOC documentation.

**Design, Setting, and Participants:**

This pragmatic, stepped-wedge cluster randomized clinical trial included patients aged 65 years or older admitted to 1 of 14 units at 2 urban hospitals in New York and Boston from July 1, 2021, to October 31, 2022.

**Intervention:**

The intervention involved PCEs (social workers and nurses trained in GOC communication) facilitating GOC conversations with patients and/or their decision-makers using a library of brief, certified video decision aids available in 29 languages. Patients in the control period received usual care.

**Main Outcome and Measures:**

The primary outcome was GOC documentation, which included any documentation of a goals conversation, limitation of life-sustaining treatment, palliative care, hospice, or time-limited trials and was obtained by natural language processing.

**Results:**

A total of 10 802 patients (mean [SD] age, 78 [8] years; 51.6% male) were admitted to 1 of 14 hospital units. Goals-of-care documentation during the intervention phase occurred among 3744 of 6023 patients (62.2%) compared with 2396 of 4779 patients (50.1%) in the usual care phase (*P* < .001). Proportions of documented GOC discussions for Black or African American individuals (865 of 1376 [62.9%] vs 596 of 1125 [53.0%]), Hispanic or Latino individuals (311 of 548 [56.8%] vs 218 of 451 [48.3%]), non-English speakers (586 of 1059 [55.3%] vs 405 of 863 [46.9%]), and people living with Alzheimer disease and related dementias (520 of 681 [76.4%] vs 355 of 570 [62.3%]) were greater during the intervention phase compared with the usual care phase.

**Conclusions and Relevance:**

In this stepped-wedge cluster randomized clinical trial of older adults, a GOC video intervention delivered by PCEs resulted in higher rates of GOC documentation compared with usual care, including among Black or African American individuals, Hispanic or Latino individuals, non-English speakers, and people living with Alzheimer disease and related dementias. The findings suggest that this form of patient-centered care delivery may be a beneficial decision support tool.

**Trial Registration:**

ClinicalTrials.gov Identifier: NCT04857060

## Key Points

**Question:**

Can implementing an evidence-based goals-of-care (GOC) video intervention delivered by palliative care educators (PCEs) in hospital settings improve GOC documentation among older patients?

**Findings:**

In this pragmatic, stepped-wedge cluster randomized clinical trial that included 10 802 adults aged 65 years or older from 14 units at 2 US hospitals, patients randomized to hospital units receiving the GOC video intervention had more GOC documentation than patients randomized to hospital units receiving usual care.

**Meaning:**

In this trial, the use of a GOC video intervention delivered by PCEs improved GOC documentation for older adults in hospital settings.

## Teaser

This stepped-wedge cluster randomized clinical trial assesses whether implementation of an evidence-based goals-of-care (GOC) video intervention delivered by palliative care educators to older adults in a hospital setting improves GOC documentation.

## Introduction

Patients communicating their goals and values to their clinicians promotes patient-centered, high-quality care.^1^ The aim of such communication is to elicit patients’ preferences and empower them to guide their care.^2^ Goals-of-care (GOC) communication is not limited to patients facing serious or life-threatening illnesses; rather, it can support adults regardless of age or health status in sharing their preferences, values, and goals for medical care.^3,4^ Goals-of-care communication has been associated with less intensive medical care near death; reductions in stress, anxiety, and depression in surviving relatives; improvements in patient and family satisfaction; and receipt of goal-concordant care.^5,6,7,8,9,10,11,12^ A failure to provide care aligned with patients’ preferences is a medical error.^1^

Despite the benefits of GOC communication, clinicians often feel poorly prepared or lack sufficient time to engage in discussions about GOC,^13,14,15^ and patients and families often have difficulty understanding such conversations.^16,17^ Traditionally, clinicians focus on medical situations rather than patient values and goals and describe hypothetical scenarios and medical interventions to explore GOC. This approach is limited because it is challenging for clinicians to portray medical interventions and their outcomes accurately and for patients to realistically envision such scenarios. The COVID-19 pandemic highlighted the urgency of improving GOC communication.^18,19^ Video decision aids that address patient and clinician barriers to GOC discussions offer a potential scalable and rapid strategy to improve GOC communication.

Our team previously developed and studied GOC video decision aids and demonstrated increases in patient communication regarding goals and preferences across clinical settings and populations as well as increases in patients receiving goal-concordant care.^11,12,20,21,22,23,24,25,26^ The videos do not replace clinical conversations; rather, they provide a common set of video depictions and a shared vocabulary to promote patient-clinician communication. The decision aids, however, have not been studied in the inpatient setting or in a manner that has been integrated with clinical staff. In response to the COVID-19 pandemic and to rapidly increase GOC documentation, we studied the implementation of a suite of videos delivered by palliative care educators (PCEs), who were social workers and nurses trained in palliative care and GOC communication, to proactively engage patients and their caregivers with the videos. We hypothesized that implementing the intervention in hospital settings would increase the rate of GOC documentation for older adults.^27^

## Methods

### Trial Design and Oversight

We conducted the pragmatic, multicenter, stepped-wedge cluster randomized Video Inspired Discussions to Improve Ethical Outcomes with Palliative Care Educators (VIDEO-PCE) clinical trial (NCT04857060) to compare PCEs delivering video decision aids and facilitating GOC communication (intervention) with usual care (control). Because the intervention included communication with staff in clinical wards, randomization was performed at the inpatient unit level.^28,29^ The stepped-wedge cluster randomized clinical trial design was chosen to facilitate implementation of the intervention in all participating inpatient units while minimizing the risk of control units’ exposure to the intervention.^30^ The trial was conducted between July 1, 2021, and October 31, 2022. A data safety and monitoring board was established and convened biannually. Institutional review board approval was secured through Boston Medical Center. A protocol outlining all trial activities was previously published,^27^ and the trial protocol is given in Supplement 1. A waiver of individual informed consent and Health Insurance Portability and Accountability Act authorization for the VIDEO-PCE intervention was granted by the Boston University Medical Campus institutional review board, acting as the single institutional review board of record for this study. The waiver was granted due to the determination that the research was not greater than minimal risk under 45 CFR §46. This study was prepared in accordance with the Consolidated Standards of Reporting Trials (CONSORT) reporting guideline.^31^

According to the stepped-wedge cluster randomized clinical trial design, each inpatient unit (cluster) began in the control phase and transitioned to the intervention phase at a randomly assigned time (wedge). Seven inpatient units were included from each of the 2 urban sites: Boston Medical Center (Boston, Massachussets) and North Shore University Hospital (Manhasset, New York). To promote covariate balance, prior to the start of data collection, each hospital unit to be included in the trial at Boston Medical Center was paired based on size and function with a matched unit at North Shore University Hospital to create 7 paired clusters. A set of uniform random numbers was generated and used to assign the order of paired clusters for intervention initiation. Following a 2-month baseline period in which no units were exposed to the intervention, a new paired cluster was exposed to the intervention every 2 months (eTable 1 in Supplement 2). Once units were exposed to the intervention, they remained exposed for the remainder of the study.

### Patients

Patients aged 65 years or older who were admitted to 1 of the 14 inpatient units for at least 8 weekday daytime hours were eligible for inclusion in the study. We chose 8 hours as a reasonable opportunity for exposure to the intervention. There were no exclusion criteria.^32^

### Intervention

The intervention involved the PCEs facilitating GOC conversations with patients and/or their decision-makers using a library of brief, certified video decision aids that addressed a range of topics (eg, GOC, cardiopulmonary resuscitation, hospice, palliative care, time-limited trials, Alzheimer disease and related dementias [ADRD], and COVID-19). The decision aids are grounded in the theory of shared decision-making^33,34^ and were designed to assist patients and decision-makers with envisioning their GOC.^11,12,20,21,23,35,36,37,38,39,40,41,42^ The decision aids were available in 29 commonly spoken languages in the New York City and Boston areas and ranged from 2 to 9 minutes in duration. The PCEs were social workers and nurses hired to coordinate with the palliative care team at each site and who received VitalTalk communication skills training via a highly structured series of video conferences.^27^

Each morning, a list of eligible patients for intervention units was generated by each site’s clinical data warehouse and provided to the PCEs, who used their clinical judgment to triage which patients they would engage. Newly admitted patients without documented GOC discussions were prioritized over patients with documented GOC recorded prior to the start of the intervention. The PCEs did not engage patients with a documented GOC discussion at admission or those who were receiving palliative care services (eFigure in Supplement 2).

The engagement of PCEs was proactive; their services were not contingent on a request for consultation from the treating practitioner. The PCEs were able to tailor the encounter and choose which video to use according to the patient’s circumstances or to not use a video for uncomplicated discussions. The PCEs showed videos on a tablet computer but were also able to share videos via text or email for remote caregivers of patients who were incapacitated (eg, patients with ADRD, patients receiving mechanical ventilation) or for patients in isolation (eg, patients with COVID-19).

The PCEs saw patients independently, engaging patients with GOC shared decision-making conversations. After interacting with patients, PCEs documented findings in the electronic health record (EHR), coordinated care with the patient’s treating team, and stimulated a palliative care consultation in complex cases.

Monthly 1-hour group sessions were conducted with the PCEs throughout the study period by 2 palliative care clinicians (J.R.L. and P.M.R.). These sessions included audit and feedback, education about clinical decision-making, review of difficult cases, and coaching.

### Outcomes and Data Collection

The primary end point was GOC documentation in the EHR during the index hospitalization for patients aged 65 years or older. We used ClinicalRegex, version 1.1.2 (Lindvall Lab, Dana-Farber Cancer Institute), a validated natural language processing (NLP) software for NLP-assisted human adjudication of GOC communication documented in the free text of clinical notes as our primary outcome, as previously reported.^43,44,45,46,47,48^ Goals-of-care communication included any documentation of a conversation about goals (including discussion of a surrogate decision-maker), limitation of life-sustaining treatment, palliative care, hospice, or time-limited trials (eTable 2 in Supplement 2). Research staff who were unaware of patients’ treatment-group assignments performed the adjudication within the software.^43^ Specifically, text blocks identified by NLP from all EHR notes for each patient were presented in the software for evaluation by trained research staff at each site. Training data sets were used to conduct ongoing quality assurance and to ensure consistent adjudication at each site.

Patient-level demographic data were derived from the EHR, and patient-level race and ethnicity data were self-reported. Race and ethnicity categories included American Indian or Alaska Native, Asian, Black or African American, Hispanic or Latino, non-Hispanic or non-Latino, Native Hawaiian or Other Pacific Islander, White, and other (Guamanian or Chamorro, Middle Eastern, and other) or more than 1 race. Race and ethnicity were analyzed because the engagement in GOC conversations is not equally distributed among racial and ethnic minority groups, and our study sought to address that disparity. A full description of the data elements collected throughout the VIDEO-PCE trial has been published elsewhere.^27^

### Statistical Power

For the primary outcome of GOC documentation, a sample size of 440 patients per wedge in a χ^2^ test for independent data provided 80% power at a 2-sided α = .05 to detect a difference in the proportion of patients with GOC documentation of 35% in the intervention group compared with 25% in the usual care group—values consistent with prior research^49^ and expectation based on clinical data from the 2 health systems. Based on our planned number of steps, enrollment per wedge, and an intraclass correlation coefficient of 0.01, the design effect used was 2.72. Thus, we needed to obtain outcome data from the records of at least 2394 patients overall (1197 per health system) to provide 80% power for our analysis of intervention effectiveness. We anticipated that records from 9000 patients would be available for analyses, with the goal of providing adequate power to test for heterogeneity of treatment effects.

### Statistical Analysis

The unit of analysis was patients, with an intention-to-treat principle used in which all patients eligible for the intervention were included independent of whether they received PCE services. Data were summarized using means (SDs) for continuous variables and counts with percentages for categorical variables. All hypothesis tests used 1-sided *P* < .05. Mixed-effects logistic regression models for correlated binary outcome data were used to estimate and test differences in the proportion of patients with GOC documentation in the intervention and usual care groups, accounting for clustering by inpatient unit. Varying secular trends across hospitals were applied for model estimation. We examined the heterogeneous treatment effects by race, ethnicity, language, and diagnosis of ADRD (determined by the presence of a prespecified “ADRD or related dementia” *International Statistical Classification of Diseases and Related Health Problems, Tenth Revision* code [eTable 4 in Supplement 2]). All data for evaluating the primary outcome were derived from the EHRs. Analyses relied on data from the patient’s index hospitalization; there was no crossover of data for patients with multiple hospital admissions. Patients who transferred between study units during their index hospitalization were assigned to contribute intervention data if they spent at least 8 daytime weekday hours in an intervention unit. We conducted all analyses using SAS, version 9.4 (SAS Institute Inc).

## Results

**Figure 1.**
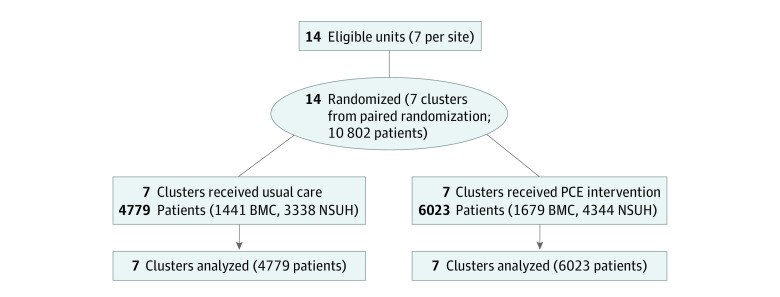
CONSORT Diagram BMC indicates Boston Medical Center; NSUH, North Shore University Hospital; PCE, palliative care educator.

During the study period, 10 802 patients (mean [SD] age, 78 [8] years; 5218 female [48.3%]; 5574 male [51.6%]) were admitted to 1 of 14 inpatient units at 2 US hospitals (Figure 1). Overall, 4779 and 6023 patients were hospitalized during the usual care and intervention phases, respectively (Table 1). Of all patients, 0.3% were American Indian or Alaska Native; 8.3%, Asian; 23.2%, Black or African American; 9.2%, Hispanic or Latino; 88.3%, non-Hispanic or non-Latino; less than 0.1%, Native Hawaiian or Other Pacific Islander; 51.8%, White, and 9.9% other or more than 1 race. Patients in the usual care and intervention groups were similar in baseline demographics (eTables 1 and 3 in Supplement 2).

**Table 1.** Characteristics of Eligible Patients^a^

Characteristic	Patients, No. (%)		
	Usual care (n = 4779)	PCE intervention (n = 6023)	Overall (N = 10 802)
Age, mean (SD), y	78 (8)	78 (8)	78 (8)
Sex^b^			
Female	2331 (48.8)	2887 (47.9)	5218 (48.3)
Male	2440 (51.1)	3134 (52.0)	5574 (51.6)
Race			
American Indian or Alaska Native	14 (0.3)	16 (0.3)	30 (0.3)
Asian	386 (8.1)	509 (8.5)	895 (8.3)
Black or African American	1125 (23.5)	1376 (22.8)	2501 (23.2)
Native Hawaiian or Other Pacific Islander	3 (0.1)	2 (<0.1)	5 (<0.1)
White	2483 (52.0)	3113 (51.7)	5596 (51.8)
Other or >1 race^c^	454 (9.5)	617 (10.2)	1071 (9.9)
Declined or missing	314 (6.6)	390 (6.5)	704 (6.5)
Ethnicity			
Hispanic or Latino	451 (9.4)	548 (9.1)	999 (9.2)
Not Hispanic or Latino	4200 (87.9)	5337 (88.6)	9537 (88.3)
Declined or missing	128 (2.7)	138 (2.3)	266 (2.5)
Language			
English	3864 (80.9)	4909 (81.5)	8773 (81.2)
Spanish	315 (6.6)	352 (5.8)	667 (6.2)
Other	548 (11.5)	707 (11.7)	1255 (11.6)
Unknown or missing	52 (1.1)	55 (0.9)	107 (1.0)
ADRD diagnosis^d^			
Yes	570 (11.9)	681 (11.3)	1251 (11.6)
No	4209 (88.0)	5342 (88.7)	9551 (88.4)

Abbreviations: ADRD, Alzheimer disease and related dementias; PCE, palliative care educator.

^a^ All data were obtained from the electronic health records at each study site. Sex, race, and ethnicity were self-reported at each site.

^b^ Ten patients (8 receiving usual care; 2, PCE intervention) had unknown sex.

^c^ Other was composed of Guamanian or Chamorro, Middle Eastern, and other.

^d^ Determined by the presence of a prespecified “ADRD or related dementia” *International Statistical Classification of Diseases and Related Health Problems, Tenth Revision* (*ICD-10*) code. A full list of *ICD-10* codes used to identify ADRD diagnoses is given in eTable 4 in Supplement 2.

### Intervention Fidelity

Each hospital had 3 PCEs over the course of the trial. The 6 total PCEs worked Monday through Friday for a mean (SD) of 4.0 (0.6) full-time equivalent per month of the intervention period. The PCEs evaluated and wrote notes on 3355 of the 6023 patients hospitalized during the intervention phase (55.7%). During the final 6 months of the trial, a mean (SD) of 70.9 (7.1) patients per month were seen and had a PCE note for each monthly full-time equivalent of PCE effort.

There were 1672 videos viewed during the intervention period among 3355 patients evaluated by PCEs (49.8%).^20,50^ Most videos (1333 [79.7%]) were viewed past the 50% mark, and 470 videos (28.1%) were viewed remotely. Of the 1672 video views, 869 (52.0%) were about GOC; 293 (17.5%), ADRD; 273 (16.3%), advance directives; and 162 (9.7%), cardiopulmonary resuscitation, intubation, and ventilatory support. Of the 1672 views, 324 (19.4%) were non-English videos.

### Outcomes

**Figure 2.**
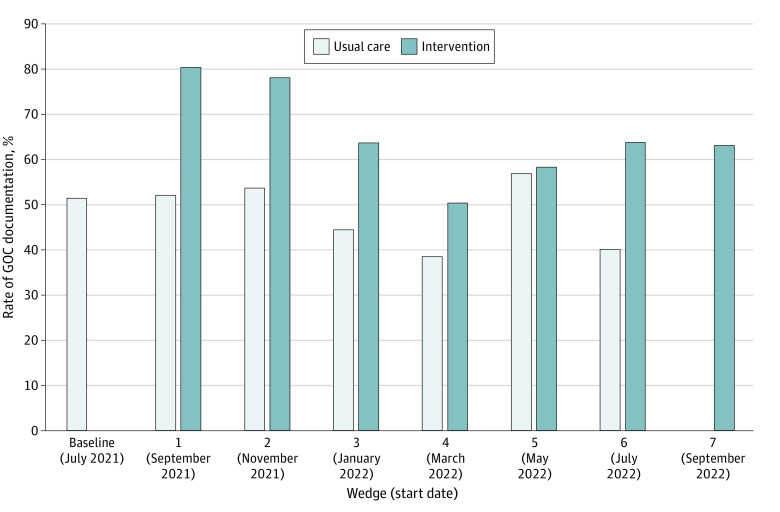
Comparison of Goals-of-Care (GOC) Documentation Rates by Cluster and Wedge The last date in the figure (September 2022) corresponds to the start date of the last 2-month intervention period of the trial. As such, the last date of the intervention was October 31, 2022.

The proportion of hospitalized patients with GOC documentation was greater for patients during the intervention phase (3744 of 6023 [62.2%]) compared with the usual care phase (2396 of 4779 [50.1%]) (*P* < .001) (Table 2). When adjusted for mixed-effects logistic regression models for hospital and secular trend, there was an increased odds of GOC documentation among intervention-phase patients (odds ratio, 3.37; 95% CI, 2.90-3.92) (Table 2). The proportions of documented conversations of goals (3562 [59.1%] vs 2258 [47.2%]; *P* < .001), limitation of life-sustaining treatment (1979 [32.9%] vs 1242 [26.0%]; *P* < .001), and palliative care (2067 [34.3%] vs 700 [14.6%]; *P* < .001) were greater for patients during the intervention phase compared with the usual care phase (Table 2). Goals-of-care documentation rate by wedge is shown in Figure 2.

**Table 2.** Comparison of Goals-of-Care Documentation During the Usual Care Phase and PCE Intervention Phase

Documentation domain	Patients, No. (%)		OR (95% CI)^a^	*P* value^b^
	Usual care (n = 4779)	PCE intervention (n = 6023)		
Overall	2396 (50.1)	3744 (62.2)	3.37 (2.90-3.92)	<.001
Goals conversation	2258 (47.2)	3562 (59.1)	3.22 (2.77-3.74)	<.001
Limitation of life-sustaining treatment	1242 (26.0)	1979 (32.9)	1.84 (1.57-2.17)	<.001
Palliative care	700 (14.6)	2067 (34.3)	4.33 (3.65-5.15)	<.001
Hospice	461 (9.6)	597 (9.9)	1.15 (0.92-1.44)	.23
Time-limited trials	20 (0.4)	105 (1.7)	1.55 (0.70-3.42)	.28

Abbreviations: OR, odds ratio; PCE, palliative care educator.

^a^ A mixed-effects logistic regression model for correlated binary data adjusted for hospital and secular trend was used.

^b^ *P* value for parameter estimates accounting for clustering by inpatient unit.

The proportions of documented GOC discussions for Black or African American individuals (865 of 1376 [62.9%] vs 596 of 1125 [53.0%]), Hispanic or Latino individuals (311 of 548 [56.8%] vs 218 of 451 [48.3%]), non-English speakers (586 of 1059 [55.3%] vs 405 of 863 [46.9%]), and people living with ADRD (520 of 681 [76.4%] vs 355 of 570 [62.3%]) were greater for patients during the intervention phase compared with usual care phase (Table 3). There was no evidence of heterogeneity of treatment effects by race, ethnicity, language, or diagnosis of ADRD.

**Table 3.** Comparison of Goals-of-Care Documentation by Race and Ethnicity, Language, and ADRD Diagnosis During the Usual Care Phase and PCE Intervention Phase^a^

Subgroup	Patients, No./total No. (%)		OR (95% CI)^b^	*P* value^c^
	Usual care	PCE intervention		
Race and ethnicity				
Asian	179/386 (46.4)	284/509 (55.8)	3.25 (1.92-5.51)	<.001
Black or African American	596/1125 (53.0)	865/1376 (62.9)	3.51 (2.57-4.81)	<.001
Hispanic or Latino	218/451 (48.3)	311/548 (56.8)	2.10 (1.33-3.32)	.002
White	1232/2483 (49.6)	2001/3113 (64.3)	3.38 (2.74-4.17)	<.001
Other or >1 race^d^	224/454 (49.3)	356/617 (57.7)	2.59 (1.59-4.20)	<.001
English speaker				
Yes	1962/3864 (50.8)	3120/4909 (63.6)	3.49 (2.96-4.13)	<.001
No	405/863 (46.9)	586/1059 (55.3)	2.52 (1.77-3.59)	<.001
ADRD diagnosis^e^	355/570 (62.3)	520/681 (76.4)	3.64 (2.24-5.90)	<.001

Abbreviations: ADRD, Alzheimer disease and related dementias; OR, odds ratio; PCE, palliative care educator.

^a^ Goals-of-care documentation is the composite of conversations about goals, limitation of life-sustaining treatment, palliative care, hospice, and time-limited trials.

^b^ A mixed-effects logistic regression model for correlated binary data adjusted for hospital and secular trend was used.

^c^ *P* value for parameter estimates accounting for clustering by inpatient unit.

^d^ Other was composed of Guamanian or Chamorro, Middle Eastern, and other.

^e^ Determined by the presence of a prespecified “ADRD or related dementia” *International Statistical Classification of Diseases and Related Health Problems, Tenth Revision* (*ICD-10*) code. A full list of *ICD-10* codes used to identify ADRD diagnoses is given in eTable 4 in Supplement 1.

## Discussion

In this pragmatic, stepped-wedge cluster randomized clinical trial that included a racially and ethnically diverse population, an intervention in which PCEs shared decision-making video decision aids and facilitated GOC communication increased the proportion of hospitalized patients with GOC documentation. Video use was robust for patients seen by PCEs, demonstrating successful implementation. The intervention was also associated with increased GOC documentation for Black or African American individuals, Hispanic or Latino individuals, non-English speakers, and patients living with ADRD.

Providing optimal serious illness care involves understanding patient values, goals, and preferences. For many medical decisions, there often is not a clear superior path for treatment and multiple reasonable options exist that require engaging patient values and preferences.^34^ Videos offer concrete images to better inform discussions and to spark GOC communication. Improving clinician communication and preserving patient autonomy have been the focus of a large corpus of research and trials since at least the time of the Study to Understand Prognoses Preferences Outcomes and Risks of Treatment (SUPPORT) in the 1990s.^51,52^ Yet, despite the professional and societal commitment^51^ toward improving GOC communication, most patients still do not communicate their preferences and values to their clinicians.^53^ The VIDEO-PCE trial offers a potential path forward.

The VIDEO-PCE trial leveraged a proactive model in which PCEs engaged all patients aged 65 years or older in GOC communication by providing them with videos that raise patients’ awareness and understanding of treatment options and possible outcomes.^34^ Instead of relying on busy clinicians or waiting for a palliative care consultation to engage in GOC communication, patients or their caregivers were engaged in GOC discussions and shared decision-making as a routine part of medical care. Importantly, PCEs documented GOC communication in the EHR and followed up directly with the clinical team. The PCE model allows for more efficient use of limited staff resources and leverages videos to empower patients or their caregivers.

A key finding of the trial was increased GOC documentation across each racial and ethnic group. Prior research suggests that Black or African American and Hispanic or Latino individuals are less likely to engage or be engaged in GOC conversations.^53,54,55^ For many individuals and especially those from racial and ethnic communities disproportionally impacted by COVID-19, GOC discussions are real and no longer hypothetical. To date, the VIDEO-PCE trial is the third published large pragmatic trial since the beginning of the COVID-19 pandemic to suggest that GOC interventions can be designed that engage Black or African American and Hispanic or Latino patients in GOC conversations.^20,56^ Due to the COVID-19 pandemic, GOC discussions may be more salient, especially for individuals from communities that have been disproportionally impacted by COVID-19.

Notably, the intervention increased GOC documentation for English and non-English speakers as well as for persons living with ADRD. The videos were available in 29 languages, 19.4% of videos viewed were in languages other than English, and 17.5% of the videos viewed were related to caregiving for a person with ADRD. Engaging non-English speakers with videos in a patient’s or caregiver’s preferred language and sharing ADRD-specific videos via text or email with caregivers of persons living with ADRD who were often remote allowed for patients and caregivers to be meaningfully engaged in a shared decision-making discussion.

It is notable that the VIDEO-PCE model not only increased GOC documentation rates overall but also did so among groups that are least likely to have documented shared decision-making conversations. While the intervention improved GOC documentation in all groups, it did not narrow underlying racial and ethnic disparities. More research is needed to identify opportunities to ameliorate racial and ethnic disparities in GOC decision-making.

### Limitations

Our findings must be considered in the context of several limitations. First, stepped-wedge designs have significant limitations (eg, partial confounding by time) and should be used sparingly and when appropriate.^30,57,58^ Although individually randomized or parallel cluster trials are frequently preferable, the circumstances of the inpatient setting (eg, shared rooms and shared staff for units) make contamination a significant concern, beyond what is tolerated in individual randomization designs.^57,59^ In response to the COVID-19 pandemic, the 2 hospitals in our study were embarking on implementing the GOC intervention but agreed to stagger the rollout in a stepped-wedge fashion to more rigorously study outcomes. Nonetheless, the relatively short wedge lengths (ie, 2 months) and robust number of clusters helped allay some of the inherent design flaws of stepped-wedge trials.^58^ Second, we looked at GOC documentation rates during the index hospitalization. Long-term studies looking at care delivery and concordance with patient goals are ongoing; however, assessing GOC documentation suggests a shared decision-making encounter, which is the gold standard for patient-centered, high-quality care.^34,60,61^ Third, we did not analyze the quality of GOC documentation during the intervention and usual care phases. Qualitative analyses from both phases are ongoing. Fourth, the intervention was designed for PCEs to approach patients for a single encounter. Longitudinal interventions are likely to have more effect and may provide the opportunity to better support patients and families with lower levels of health literacy or trust and may be the key to managing racial and ethnic disparities. Fifth, this intervention was conducted with hospitalized patients; moving the intervention to the outpatient setting may provide a milieu that may be more effective, not influenced by an acute decompensation, and more amenable to longitudinal engagement. Sixth, the videos used in this intervention are intended to be support tools used in clinical encounters. Accordingly, we were not able to differentiate the effect of the videos from that of the PCEs.

## Conclusions

The VIDEO-PCE randomized clinical trial demonstrated a significant and clinically meaningful increase in GOC documentation rates among hospitalized older adults receiving a GOC video intervention delivered by PCEs, a scalable intervention that could be quickly implemented across many hospitals, compared with usual care. Notably, the intervention resulted in an increase in GOC documentation for Black or African American individuals, Hispanic or Latino individuals, non-English speakers, and persons living with ADRD. Such benefits have been elusive. This trial found a benefit of a proactive model of care with social workers and nurses to facilitate GOC communication with video decision support tools that were available in 29 languages. To date, the VIDEO-PCE intervention represents one of the first rapidly adoptable paradigms with a clinically meaningful increase in GOC documentation, a widely used quality metric that reflects high-quality, patient-centered care delivery.

## Notes

Supplement 1.
Trial Protocol

Supplement 2. **eFigure.** Protocol for PCE Patient Interaction **eTable 1.** VIDEO-PCE Trial Intervention Timeline **eTable 2.** Natural Language Processing Keyword Library **eTable 3.** Primary Discharge Diagnosis of Eligible Patients **eTable 4.** List of *ICD-10* Diagnosis Codes Used to Identify ADRD Patients

Supplement 3.
Data Sharing Statement
