# Supplementary material for: Video Intervention and Goals-of-Care Documentation in Hospitalized Older Adults: The VIDEO-PCE Randomized Clinical Trial
Source: JAMA Netw Open. 2023 Sep 11;6(9):e2332556. doi: 10.1001/jamanetworkopen.2023.32556 (PMC10495866; doi:10.1001/jamanetworkopen.2023.32556)
Supplement: Supplement 1. — Trial Protocol [file jamanetwopen-e2332556-s001.pdf]

# Meeting the Challenges of COVID-19 by Expanding the Reach of Palliative Care: Proactive Advance Care Planning with Videos for the Elderly and all Patients with Dementia

**ClinicalTrials.gov number:** NCT04857060
**BMC Protocol Number:** H-41482
**Protocol Version Number:** 1.2
**Protocol Version Date:** October 7. 2022
**Funding Mechanism:** NIH/NIA 1 R01 AG072911
**Principal Investigator:** Michael Paasche-Orlow, MD, MPH
**Phone:** 617-414-6936
**E-mail:** mpo@tuftsmedicine.org

## TABLE OF CONTENTS


1 List of Abbreviations ... 3
2 Protocol Summary ... 3
3 Background/Rationale & Purpose ... 4
3.1 Background Information ... 4
3.2 Rationale and Purpose ... 5
4 Objectives ... 6
4.1 Study Objectives ... 6
4.2 Study Outcome Measures ... 7
4.2.1 Primary Outcome Measures ... 7
4.2.2 Secondary Outcome Measures ... 7
5 Study Design ... 8
6 Potential Risks and Benefits ... 9
6.1 Risks ... 8
6.2 Potential Benefits ... 9
6.3 Analysis of Risks in Relation to Benefits ... 9
7 Study Subject Selection ... 9
7.1 Subject Inclusion Criteria ... 9
7.2 Subject Exclusion Criteria ... 10
8 Study Intervention ... 11
9 Study Procedures ... 11
10 Assessment of Safety and Data Safety Monitoring Plan (DSMP) ... 17
10.1 Definitions ... 17
10.2 Safety Review ... 18
10.3 Reporting Plans ... 19
10.4 Stopping Rules ... 19
11 Data Handling and Record Keeping ... 17
11.1 Confidentiality ... 17
11.2 Case Report Forms ... 20
11.3 Study Records Retention ... 19
11.4 Data Management ... 21
12 Statistical Plan ... 22
12.1 Study Hypotheses ... 22
12.2 Sample Size Determination ... 23
12.3 Statistical Methods ... 23
13 Ethics/Protection of Human Subjects ... 27
14 Literature References ... 28

# 1 List of Abbreviations

| Abbreviation | Abbreviation definition |
|---|---|
| ACP | Advance Care Planning |
| ADRD | Alzheimer's Disease and Related Dementias |
| CPR | Cardio-Pulmonary Resuscitation |
| PCE | Palliative Care Educator |
| SW-CRT | Stepped Wedge Cluster Randomized Trial |
| BMC | Boston Medical Center |
| NLP | Natural Language Processing |
| EHR | Electronic Health Record |
| RA | Research Assistant |

# 2 Protocol Summary

| | |
|---|---|
| **Title:** | Meeting the Challenges of COVID-19 by Expanding the Reach of Palliative Care: Proactive Advance Care Planning with Videos for the Elderly and all Patients with Dementia |
| **Short Title:** | Video Images about Decisions to Improve Ethical Outcomes with Palliative Care Educators (VIDEO-PCE) |
| **Population:** | Aim 1: Patients ≥65 years admitted to one of the study inpatient units<br>Aim 2a: Any patient ≥18 years admitted to one of the study inpatient units with ADRD/delirium<br>Aim 2b: Adult Caregiver of any patient in aim 2a |
| **Intervention:** | Palliative Care Educator using video decision aid shown at time of admission to patients/caregivers in wards/units randomized to the intervention phase |
| **Objectives:** | The **overall objective** of the present proposal is to reduce the burden of COVID-19 and advanced illness and its consequences for an aging U.S. population. |
| **Design/Methodology:** | This project is a multi-center stepped wedge cluster randomized trial of an advance care planning (ACP) video intervention (vs. standard of care) using a Palliative Care Educator among patients ≥65 years OR any patient ≥18 years old with ADRD regardless of age admitted to one of the study inpatient units |
| **Total Study Duration:** | 2 years |
| **Subject Participation Duration:** | EHR data collection during the 16 months of enrollment (2 months baseline plus 14 months intervention steps). EHR data abstracted for one year after the end of the 16 months of enrollment. |

# 3 Background/Rationale & Purpose

## 3.1 Background Information

COVID-19 disproportionately affects the elderly and those with Alzheimer's Disease and Related Dementias (ADRD/Delirium).[1, 2] The COVID-19 pandemic has killed over 500,000 Americans and is a common and morbid condition, especially in people over the age of 65 and those with functional impairment and ADRD/Delirium.[2] When COVID-19 strikes, these patients die at higher rates.[2, 3] The surge in patients with COVID-19 poses a significant public health challenge and has the potential to compromise the orderly function of health institutions.[4]

The palliative care needs of inpatients with ADRD/Delirium and those over 65 is rapidly increasing, and access to palliative care clinicians is limited. The majority of hospitals in the U.S. have palliative care programs; indeed, over the past decade access to palliative care services has increased dramatically.[5-9] However, staffing capacity to meet the clinical needs continues to be a significant challenge.[10-13] Many hospitals provide palliative care services only to a small portion of the patients that would be appropriately served by palliative care[14, 15] and the scope of this problem has increased with COVID-19. New models are needed for palliative care services to meet the need.[16] Older patients and those with ADRD/Delirium face the prospect of receiving burdensome and unwanted end-of-life care due to lack of palliative care services.

Decision making in patients with ADRD/Delirium and their caregivers during COVID-19 is urgent. Patients with ADRD/Delirium have a small window of opportunity to state their preferences for the advanced stages of the disease before their disease makes them incapable of decision making. Without an Advance Care Planning (ACP) discussion, caregivers are often left to make treatment decisions for their loved ones with the advanced stages of the disease.[17] Numerous studies have shown that caregiver decision making is no better than chance and often lacks stability over time.[18] Caregivers often suffer a great deal of burden and distress attempting to develop a comprehensive care plan for the advanced stages of the disease.[17] Caregiver stress and communication challenges are exacerbated by their exclusion from the hospital.[19-21] COVID-19 poses significant ACP challenges for patients with ADRD/Delirium and their caregivers.

This study will be conducted in compliance with the protocol, applicable regulatory requirements, and BMC/BU Medical Campus Human Research Protection policies and procedures.

## 3.2 Rationale and Purpose

Advance care planning (ACP) in older patients or patients with ADRD/Delirium needs improvement: ACP seeks to ensure that patients receive medical care consistent with their values, goals and preferences during serious and chronic illness.[22] The lack of ACP documentation is associated with greater use of aggressive interventions, more terminal hospitalizations, lower hospice use, higher health care costs, and worse family bereavement

outcomes.[17, 22] Unfortunately, ACP documentation in older patients and patients with ADRD/Delirium remains inadequate.[23, 24] Furthermore, marked racial and regional disparities persist in ACP documentation for seriously ill patients.[25] For the ACP process to lead to optimal decisions, patients and their caregivers require accurate, impartial and comprehensible information about their treatment options, and a care setting where communication needs are addressed early in their illness by a dedicated clinician.[26-28] However, studies show that traditional written and verbal ACP does not effectively inform many patients and caregivers, and often occurs late in the disease process.[22] High-quality ACP increases patient safety by ensuring that patients receive effective care that meets their goals.

Video decision support improves ACP: The traditional approach to ACP, which primarily relies on ad hoc verbal descriptions of hypothetical clinical situations and treatment choices, is limited because complex scenarios are difficult to envision, provider information is inconsistent, and verbal explanations are hampered by literacy, emotional and language barriers.[22, 29-31] Over the past few years, investigators have recognized the shortcomings of prior efforts and have developed new interventions to better facilitate ACP.[22, 32-37] The video intervention proposed for this study focuses on patient, caregiver and clinician communication about treatments for medical care facilitated by a Palliative Care Educator (PCE).

The COVID-19 PCE video intervention proposed for this study focuses on patient/caregiver and clinician communication about goals of care. Video aids to better educate and inform decision making are commonly used. These videos attempt to overcome language and literacy barriers and to present potential scenarios with a sense of reality lacking in verbal descriptions.[54-56] These videos are available in 25 different languages, and attempt to overcome literacy barriers and to present potential scenarios with a sense of reality lacking in verbal descriptions. In addition to using videos, our PCEs will be trained in the Vital Talk program, the most widely disseminated teaching method that focuses on patient-centered serious illness communication skills training. To our knowledge, this is the first trial of PCEs trained in communication skills to engage patients with palliative care services with videos. If effective, this model can be rapidly disseminated to improve care for millions of Americans.

Hospitalized patients often receive burdensome interventions as the default option, without a shared decision-making conversation or awareness of more comfort-oriented care.[57, 58] Thus, patients are at high risk of receiving poor-quality care at the end of life given the burden of such care on patients. Poor ACP and communication about patients' preferences for end-of-life care contribute substantially to the receipt of aggressive, costly, and unwanted medical care for patients with serious illness.[31, 46, 48, 59-62] Therefore, improving palliative care services may prove to be an effective strategy to enhance the delivery and quality of medical care for hospitalized patients. ACP video tools have shown promising efficacy in educating patients about their options and informing their preferences for care.[55, 63-66] Given the intensity of health care utilization for hospitalized patients, patients may greatly benefit from a PCE-led video intervention to expand the reach and impact of palliative care to inform and empower patients and their caregivers in the decision-making process and to improve the delivery of care that is concordant with their wishes during COVID-19.

# 4 Objectives

## 4.1 Study Objectives

The **overall objective** of this study is to reduce the burden of COVID-19 by expanding the reach of inpatient palliative care services, especially for patients with ADRD/Delirium. We propose to conduct a stepped wedge cluster randomized trial (SW-CRT) of a PCE video intervention among hospitalized patients aged 65 and over, or any patient ≥18 years with ADRD/Delirium and their caregivers in the ward and ICU settings of two major hospitals: Boston Medical Center (BMC) and North Shore University Hospital. Patient outcomes will be abstracted from electronic health records with Natural Language Processing (NLP).

We will test our hypotheses via the following **Specific Aims**:

**Aim 1:** To test the effects of a PCE video intervention leveraging video decision aids on the quality of end-of-life care. We will conduct a SW-CRT to evaluate intervention effectiveness by comparing the following outcomes among 9,000 hospitalized patients: ACP documentation; preferences for resuscitation; palliative care consults; and, hospice use. ***Hypotheses**: A higher proportion of patients in the intervention phase (vs. control) will: (1) complete advance care plans **(primary outcome),** (2) have documented resuscitation preferences, (3) have palliative care consults, (4) enroll in hospice over the course of one year of follow-up, and (5) have documented health care proxies.*

**Aim 2a:** The manual chart review activity is intended only for the patients whose caregiver participated in the survey activity. It is distinctive from the NLP activities described in our protocol which identify ACP documentation from the free-text of clinical notes. The chart review will involve a thorough human review of structured ACP elements such as DNR/DNI order, MOLST/POLST and Health Care Proxy form completion in each patient's chart.
**Aim 2b:** To characterize caregiver-centered outcomes of patients with ADRD/Delirium, including: (1) knowledge, (2) confidence in future care, (3) communication satisfaction, (4) decisional satisfaction, and (5) decisional conflict in 600 caregivers of patients with ADRD/Delirium admitted to the hospital. ***Hypothesis**: Intervention phase caregivers of patients with ADRD/Delirium (vs. control) will have higher knowledge, confidence, communication satisfaction, decisional satisfaction, and lower decisional conflict.*

**IMPACT:** COVID-19 poses a unique dilemma for older Americans and patients with ADRD/Delirium and their caregivers, who must balance their desire to live against the risk of a lonely and potentially traumatic hospital death. Video decision support is a practical, evidence-based, and innovative approach to assist patients facing such choices. We have a highly experienced team and infrastructure at BMC and North Shore to execute this proposal. If proven

effective, this innovative care model can be immediately deployed across the country to improve the quality of care for millions of Americans. Given the urgency of the need for scalable interventions, this study will provide the evidence quickly and efficiently to improve care rapidly across the country.

## 4.2 Study Outcome Measures

### 4.2.1 Primary Outcome Measures

The primary outcome of this trial is ACP documentation any time during the index hospitalization as ascertained by NLP-assisted EHR review for any qualifying documentation of ACP in the EHR note (goals of care, advance directive, MOLST/POLST, code status, palliative care or hospice) (yes versus no).

### 4.2.2 Secondary Outcome Measures

Secondary outcomes include:

- Code status preferences (Aim 1)
- Use of palliative care consult/services (Aim 1)
- Hospice use (Aim 1)
- Health Care Proxy (Aim 1)

Secondary outcomes related to Aim 2a (patients 18+ years admitted to one of the study inpatient units with ADRD/delirium) include:

- Documentation of ACP Preferences in Electronic Health Record

Secondary outcomes related to Aim 2b (caregivers of patients with ADRD/Delirium) include:

- ACP knowledge (Aim 2)
- Confidence in future care (Aim 2)
- Communication satisfaction (Aim 2)
- Decisional satisfaction (Aim 2)
- Decisional conflict (Aim 2)

# 5 Study Design

This is pragmatic SW-CRT of a PCE-led, video-assisted COVID-19 ACP intervention in inpatient-based units (3 medical-surgical wards, Medical ICU, Cardiac ICU, Neurology ICU, step-down unit) at two hospitals: BMC and North Shore University Hospital (Northwell Health). All inpatients ≥65 and all patients with ADRD/Delirium ≥18who are hospitalized on a unit during the intervention phase will receive the intervention.

This 2-year study (2 month data collection and tool preparation, staff training and site standardization (we are already embedded in both health systems doing similar work; thus this

short timeframe is feasible); 16 months rolling recruitment and surveying; 2 months data cleaning and analysis, and 4 months manuscript preparation and dissemination of findings) will roll out the intervention to 14 randomized inpatient units at 2 sites. Every two months, an additional inpatient unit will be added to our intervention at each hospital, i.e., there will be seven waves or "steps"; for a total of 7 units at each hospital.

Consistent with a SW-CRT with two hospital units per step (cluster), prior to the collection of any data in the pre-intervention period, we will generate a set of uniform random numbers for each of the seven clusters to be assigned to a starting period for the study intervention. There will be eight study periods in total with a usual care period at the start of the study for all clusters. The first randomized intervention period will then begin in period two.

| | Baseline | 14 Months of Clustered Intervention Expansion | | | | | | |
|---|---|---|---|---|---|---|---|---|
| Cluster | M0 | M2 | M4 | M6 | M8 | M10 | M12 | M14 |
| 1 | | | | | | | | |
| 2 | | | | | | | | |
| 3 | | | | | | | | |
| 4 | | | | | | | | |
| 5 | | | | | | | | |
| 6 | | | | | | | | |
| 7 | | | | | | | | |

The data needed to assess the outcomes for all patients will be derived from each hospital's EHR (Aim 1).

For Aim 2b, 600 caregivers of patients with ADRD/Delirium (300 control phase; 300 intervention phase) will be surveyed by telephone during (or within one week of) the index hospitalization to assess caregiver-centered outcomes.

Each day PCEs, who will be nurses or social workers on the palliative care team, will approach patients who are currently hospitalized under Aim 1 or Aim 2a. The PCE will then proactively use the goals-of-care video decision aid (or any of the additional videos regarding CPR, hospice, dementia, etc., as relevant and in the appropriate language) to provide educational support and assist in delivering primary palliative care services relating to goals-of-care conversations and ACP documentation. The videos range from 4-6 minutes in length and the PCE will watch the videos together with the patient and caregiver on an iPad (or remotely via telehealth with the caregiver).(130)

The PCE will arrange all video showings to include patient and caregiver (when possible and acceptable to the patient); when patients are unable to view a video (e.g., loss of capacity, delirium), the caregiver will view the video. The videos do not replace clinician counseling; indeed, they are designed to allow the PCE to confirm comprehension and to stimulate

conversation with a shared vocabulary. The PCE will then communicate the patient's or caregiver's wishes to the treating primary medical team to coordinate care.

In cases when the PCE deems that engagement with the full palliative care team is warranted, they will approach the treating primary medical team to place the consult. If/when the PCE exhausts the automated list for patients, they will coordinate with the palliative care consult team to select patients from the list of requested consultations. The PCE role will be fully integrated into existing hospital practices at our sites consistent with the pragmatic nature of this study design.

For Aim 1, the study population will consist of patients 65 years or older who are admitted to one of the study inpatient units in the hospital. For Aim 2a, the study population will consist of any patient ≥18 years admitted to one of the study inpatient units in the hospital with a diagnosis of ADRD/Delirium. For Aim 2b, the study population will consist of adult (≥18) caregivers of patients identified in Aim 2a. These caregivers will be recruited to complete a phone survey for our secondary caregiver outcomes

The data needed to assess the outcomes for all patients aged 65 or over will be derived from each hospital's EHR (Aim 1). For Aim 2, 600 caregivers (300 control phase; 300 intervention phase) will be surveyed in-person (or remotely) during the index hospitalization to assess caregiver-centered outcomes.

# 6 Potential Risks and Benefits

## 6.1 Risks

The potential risks are minimal given the fact that the intervention promotes learning about medical care for patients, improves communication for patients and their families regarding advance care planning and self-determination, and the concordance between patient's wishes and the care they receive.

The major potential risk for subjects is loss of confidentiality. Loss of confidentiality is very unlikely because specific procedures have been implemented by the research team to prevent such disclosure and these measures will be maintained during the proposed study. Another risk in Aim 1 is being upset by the intervention videos/questions. Probability of this occurrence is minimal. We have conducted a series of clinical trials for patients with advanced illnesses and have rarely had patients get upset due to the topic. In each of these cases, the participant was interested in continuing after a short break.

For subjects enrolled in Aim 2, there is a risk that they could become upset or saddened by some of the survey questions.

## 6.2 Potential Benefits

This study provides no direct benefit to subjects, however there is the potential for patients and clinicians in the clinics to benefit from the study by having their treatments better aligned with their preferences.

## 6.3 Analysis of Risks in Relation to Benefits

The minor risks for the participants in this study may be considered counterbalanced by the potential direct benefits and knowledge gained. The results gleaned from the study are intended to improve the ACP of the overall inpatient population, and particularly those with ADRD/Delirium. Thus, the risk/benefit balance for this study appears favorable.

# 7 Study Subject Selection

## 7.1 Subject Inclusion Criteria

Over the two years of the trial, we will examine data on approximately 15,000 patients ≥65 admitted to these 23* units for our primary and secondary outcomes (Aim 1). Given the pragmatic nature of this trial, our inclusion criteria are quite broad and consistent with the goal of pragmatic trials.

We will also survey caregivers of 600 patients with ADRD/Delirium to conduct a telephone administered survey for caregiver-centered outcomes (knowledge, confidence, communication satisfaction, decisional satisfaction, and decisional conflict) during the index hospitalization. Caregivers may or may not be designated as ADRD/Delirium legal surrogate decision maker for the patient (i.e., most patients with ADRD/Delirium do not have a legally designated representative.) Any adult identified in the EHR as the contact family member or friend will be eligible to partake in the caregiver survey. Half of surveys will be conducted during the control period; half during the intervention period. Caregivers will be either English- or Spanish-speaking adults, which are the languages in which our surveys are validated. For patients in the control group, surveys will be completed during the hospital stay or within 1 week of discharge. For patients in the intervention group, the survey will be completed AFTER the PCE intervention, and up to 1 week after discharge.

*14 units (7 per hospital) will be included in the stepped wedge trial, an additional 9 units (3 at BMC and 6 at NorthShore) will be used to recruit additional control participants only. These additional units were added due to low recruitment at the start of the study and the decreasing number of control units as the stepped wedge design progresses. The target enrollment of 600 caregivers is still accurate.

Adding delirium to the list of eligible diagnoses will also increase the potential number of eligible subjects. The surveys are applicable to the caregiver of any patient who is not capable of making their own health decisions; this includes patients who are experiencing any sort of memory or cognitive decline.

## 7.2 Subject Exclusion Criteria

For Aim 1, there are no exclusion criteria.

For Aim 2a, there are no exclusion criteria.

For Aim 2b, not speaking English or Spanish, which are the languages in which our surveys are validated.

- We will not be including individuals who are not yet adults (infants, children, teenagers)
- 
- We will not be including prisoners

# 8 Study Intervention

A palliative care trained provider (a nurse or social worker on the palliative care team) will serve as the PCE. Using the ACP videos on a tablet via a Video App, the PCE will provide educational support and assist in delivering primary palliative care services relating to in-the-moment goals-of-care conversations and ACP documentation for patients that are hospitalized. PCEs will be members of the Palliative Care team, coordinate daily activities with the team, and report to the head of the Palliative Care service. PCEs will serve in a triage function to manage cases that can be handled with educational support for goals-of-care conversations and ACP documentation or to stimulate full palliative care consultation. PCEs will directly coordinate communication of the patient's preferences with the treating primary medical team. A key aspect of this trial design is the fully integrated role into existing hospital practices of the PCE position.

PCEs will receive Vital Talk intensive communication skills training via a highly structured series of Zoom conferences. The PCEs will also be trained on use of the ACP certified videos using the ACP App. Training will instruct clinicians on how to: 1. Introduce the videos to patients and caregivers; 2. Use the videos as adjuncts to ACP counseling by clinicians; 3. Select the appropriate video(s) from the entire suite according to patients' needs; and, 4. Prescribe videos for patients and caregivers using the electronic platform. The suite of ACP videos is designed to address common ACP decisions confronting patients at risk or with COVID-19 and their caregivers. The videos also cover all of the decision points surrounding ADRD/Delirium (e.g., feeding tubes, resuscitation, etc.). The videos are intended to be an adjunct to clinician counseling, not to replace it. Suggested videos for clinicians to use with patients will include goals-of-care videos, general ACP videos, intervention-specific videos such as ventilatory support or CPR, and hospice videos. PCEs will also have an array of videos to support caregivers, including videos regarding compassionate extubation if this is relevant.

The PCE will encourage the patient to make their wishes known to their family or other caregiver (and will offer to facilitate a call/video-call) and the attending, and that with the patient's

permission, will relay their wishes to the treating team in addition to completing ACP documentation in the EHR. As an integrated part of existing hospital practice, PCE will communicate with the primary treating team and the palliative care team. When there are palliative care needs beyond ACP (e.g., symptom control) or if the PCE determines that the support of the full palliative care team is warranted, the PCE will recommend to the treating team to place the consult request. The PCE will not be collecting any data for research purposes only. For QI purposes, the Palliative care consult team will keep tracking documents of the PCE activities (number of patients seen per day, amount of time spent with each patient, etc). This is needed for supervisory purposes. These may be reviewed retrospectively by the research team and compared to research data. In this case, an amendment will be submitted to the IRB to cover these activities.

# 9 Study Procedures

Eligibility

Aim 1 and Aim 2a: Each day, PCEs will review a list of inpatients who are ≥65 or have a diagnosis of ADRD/Delirium

Aim 2b: Study staff will contact identified adult caregivers by phone to describe the survey activity

Recruitment

For Aim 1, all patients over the age of 65 will be included in the trial. For those patients admitted to wards/units in the intervention phase, the PCE will proceed with primary palliative care and view the ACP videos with the patients and family. For patients over the age of 65 admitted to wards/units that are in the control phase, usual care will proceed without the use of the PCE.

For Aim 2a, we will identify 600 inpatients to the study units with ADRD/Delirium who are 18+ years old.
For Aim 2b, 300 caregivers of the patients identified in Aim 2a will be surveyed during the control phase, and 300 caregivers will be surveyed during the intervention phase. For the group of caregivers (N=600) being surveyed for caregiver-centered outcomes, individual verbal informed consent will be obtained. The RA will survey caregivers using a validated survey tool. For patients with ADRD/Delirium and their caregivers that are admitted to a ward/unit that has been randomized to the intervention, the PCE will proceed with the video intervention.

Recruitment efforts for the caregiver survey (Aim 2) will be limited to English- and Spanish-speaking caregivers who are able to independently consent to participate in a research study. Research staff will work with the care team on the inpatient units where identified patients are currently hospitalized. The health care team will locate (either in person or by phone) designated caregivers for identified patients and invite them to participate. A member of the care

team will approach the caregiver and ask if they would be interested in getting a phone call from the research team to get more information about the study. If the caregiver says yes, the RA will call them, read the recruitment script (attached), and, if the subject is willing, review the consent form and complete the survey. If the designated caregiver cannot be located to invite to participate, an invitation letter with opt-out postcard will be mailed to them.

Informed Consent

For Aim 1, there are special informed consent considerations in this pragmatic SW-CRT: the hospital clinical unit is the level of randomization, the intervention is of low risk and will be implemented as the standard of care for the whole clinical unit, and data for our primary outcome and related outcomes derived from the EHR are ascertained from existing sources. Thus for this aspect of our proposal, we will seek a waiver of individual informed consent and HIPAA authorization after careful review of the criteria to do so as we have previously done successfully in prior studies. The research involves no more than minimal risk to the subjects as described above. We do not believe the waiver will adversely affect the rights and welfare of the subjects. As a pragmatic trial of thousands of hospitalized patients and clinicians, this research could not practicably be carried out without the waiver nor without access to and use of PHI of patients.

For Aim 2a, we will seek a waiver of individual informed consent and HIPAA authorization due to the fact that this is a low risk activity (chart review), and the target population is incapable of consent due to a diagnosis of ADRD/Delirium.

For Aim 2b, verbal informed consent will be obtained for the phone survey. The caregiver survey should take less than fifteen minutes.

Natural Language Processing (NLP) Data Collection

Over the course of the study, we will review the charts for all enrolled participants. For Aim 1 and Aim 2a, the inpatient EHR records (including those with ADRD/Delirium) will be analyzed (approximately 15,000 patients across both sites).

Initial NLP analyses will be done locally at each data collection site (BMC and Northwell) using software that was developed at DFCI for this purpose. The results of that initial analysis will be coded and sent as a HIPAA LDS via HIPAA approved cloud folders such as Box.com to our NLP partners at DFCI. Each site will retain a local mastercode file that will not be shared with anyone outside the institution. Patients will be assigned a unique identifier that will be used on datasets shared with DFCI.

DFCI will have a reliance agreement in place with BUMC and Data Transfer Agreements in place with both BMC and Northwell.

Every 2 months (the size of each step; 1 baseline + 7 steps = 8 data transfers) the outcomes of interest using NLP will be transferred to our data collection site (DFCI). Data on the following outcomes will be collected until the end of the study period.

- ACP discussion (e.g., goals-of-care discussion, advance directive, MOLST/POLST, code status, etc.)
- Resuscitation preferences
- Palliative care consults
- Hospice Use
- Health care proxy discussion

As stated above, direct identifiers will be held at each respective site (BMC researchers can see BMC identifiers but not North Shore University Hospital's identifiers, and vice versa.) The risks will be minimal as the data will be stored and analyzed on a HIPAA secure cluster at each site. None of the data will be stored in paper form. The data and identifiers will be kept for seven years after the end of the study period on the HIPAA secure cluster computer at each site. After the seven years, all HIPAA identifiers and all linking codes will be permanently destroyed in accordance with regulation.

<u>NLP Validation</u>

Prior to the use of NLP for outcome assessment in this trial, we will entrain and validate the NLP process for each of our two study sites.  Specifically, we will use historic note data from a sample of 20 patients from each site who meet enrollment criteria. We will then measure the validity of this process by comparing results from human assisted NLP to a human chart review. The goal of the keyword library validation process is to ensure that the keyword library and abstraction guidelines accurately represent the language used to communicate information associated study outcomes. The semi-automated note annotation process will be cross-validated across both sites. Clinical notes will be the substrate of this process and must be requested from each site's clinical data warehouse.

Each site will ensure that the appropriate IRB and Data Sharing protocols are in place before this activity begins.

Chart Review

EHR data will be extracted by manual chart review on all Aim 2a patients whose caregiver completes a survey (2b). This survey will be limited to the inpatient stay that generated the survey, and include the following elements for collection of secondary outcomes:

- DNR/DNI order
- MOLST/POLST filed
- Resuscitation/Intubation preferences
- Health Care Proxy specified
- Palliative Care Consult during hospitalization

- Discharge Disposition

A detailed chart review instrument is attached to the protocol.

<u>Withdrawal</u>
We do not anticipate any circumstances where the caregiver will withdraw from participation in the study. Study staff will make clear to the caregiver that participation is entirely voluntary and may be withdrawn at any time.

<u>Masking</u>
Due to the nature of the intervention, participants and staff will not be blinded to the intervention.

The NLP outcomes adjudication process is not fully automated in this study. We are doing a human-assisted NLP process in which a staff member validates the text presented in the software as a possible outcome. For NLP analysis, the following steps will be taken to ensure blinding to study step assignment by the staff member doing the NLP outcome attribution:

- Prior to adjudication activities, names will be coded
- Annotation will be performed in large batches with all patients enrolled who have clinical notes to that point.
- NLP notes for adjudication will not be grouped by Study ID when presented to annotators. Each note will be annotated individually, without reference to concepts contained in other notes annotated before or after.
- When possible, a staff member who did not enroll the participants will perform the annotation.

<u>Caregiver Surveys</u>
Caregivers will be surveyed using a REDcap survey. The REDCap project will be hosted by BUMC, both sites will enter data into the same project. All subjects will be assigned a unique identifier that will be entered into REDCap. All other PHI will be retained in a linking file that is not shared outside the institution.

Caregiver data will be linked with the associated patient EHR data, but this linking file will be kept locally, and only HIPAA LDS will be shared with other sites included on this protocol.

<u>Costs/Payment</u>
There are no costs to subjects for participating in this study.

Caregivers will be compensated $50 for completing the survey.

# 10 Assessment of Safety and Data Safety Monitoring Plan (DSMP)

## 10.1 Definitions

The following definitions will be used in the assessment of safety:

ACP is a standard part of clinical care for patients. The caregiver survey, however, is purely a research activity. We have had excellent experiences with prior caregiver surveys. At the same time, it is possible that this survey could make these subjects upset as they consider advance care planning issues for their family member. In the context of this study, an expected adverse event would be if the participant became distraught during the survey administration, to the point of not being able to complete the survey, or asking to end the survey prematurely. In this unlikely event, the event will be documented on an AE Reporting Form and reported per the guidelines outlined below. We do not anticipate any Serious Adverse Events.

*Unanticipated Problem* is defined as an event, experience or outcome that meets **all three** of the following criteria:

- is unexpected; AND
- is related or possibly related to participation in the research; AND
- suggests that the research places subjects or others at a greater risk of harm (including physical, psychological, economic, or social harm) than was previously known or recognized.

*Possibly related* means there is a reasonable possibility that the incident, experience, or outcome may have been caused by the procedures involved in the research

*Unexpected* means the nature, severity, or frequency of the event is not consistent with either:

- the known or foreseeable risk of adverse events associated with the procedures involved in the research that are described in (a) the protocol–related documents, such as the IRB-approved research protocol, any applicable investigator brochure, and the current IRB-approved informed consent document, and (b) other relevant sources of information, such as product labeling and package inserts; or
- the expected natural progression of any underlying disease, disorder, or condition of the subject(s) experiencing the adverse event and the subject's predisposing risk factor profile for the adverse event.

## 10.2 Safety Review

Both the risks listed in Section 6.1 and unknown risks will be monitored as follows: Participants will be informed that they may decline to answer any question that makes them feel uncomfortable. If any adverse events occur, the Principal Investigator will be notified as soon as possible and a corrective plan will be developed and put to use. All AEs will be reported to the IRB at each continuing review.

## 10.3 Reporting Plans

The Principal Investigator at the site where the event occurred will report Unanticipated Problems, safety monitors' reports, and Adverse Events to the local IRB in accordance with IRB policies:

- Unanticipated Problems involving a fatal or life-threatening event will be reported to the local IRB and to the IRB of record (BMC/BUMC IRB) within 2 days of the site Principal Investigator learning of the event.
- Unanticipated Problems not involving a fatal or life-threatening event will be reported to the local IRB within 7 days of the site Principal Investigator learning of the event.
- Reports from safety monitors with recommended changes will be reported to the IRB within 7 days of the investigator receiving the report.
- Adverse Events (including Serious Adverse Events) will be reported in summary at the time of continuing review, along with a statement that the pattern of adverse events, in total, does not suggest that the research places subjects or others at a greater risk of harm than was previously known.
- Reports from safety monitors with no recommended changes will be reported to the IRB at the time of continuing review.
- When reporting to the local IRB the site Principal Investigator will also report to the administrative study Principal Investigator Dr. Rao. Such reporting will also include all findings and determinations made by local IRBs.

The Principal Investigator will report Unanticipated Problems and Adverse Events to the Data Safety Monitoring Board at each bi-monthly Board meeting or as established in the DSMB charter.

The Data Safety Monitoring Board will communicate its reports and recommendations per IRB policies, the DSMB charter, and the study Sponsor.

Per the DSMB charter, the Board will meet every six months to review safety issues and study progress.

### 10.4 Stopping Rules

The study has no preset stopping rules.

## 11 Data Handling and Record Keeping

### 11.1 Confidentiality

Aim 1 Data: Each site (BMC and North Shore Hospital) will collect identifiable EHR data for all eligible patients as described above. All patients will be assigned a unique identifier, and each site will retain a linking file that will not be shared outside of the institution, and will only be accessible to authorized study personnel. At BMC, all data will be stored in password-protected

files on a network server located inside the BMC firewall, which is in compliance with data storage requirements for PHI as defined by BMC. At North Shore, all data will be stored on an excel spreadsheet that is password protected on Microsoft OneDrive. OneDrive is a HIPAA compliant platform for data storage and sharing and has been vetted by Northwell Health's Research IT and Research Compliance teams.

At both sites, any paper records containing study data will be stored in a locked cabinet that is only accessible by the study team. Research participants will be given unique study IDs upon enrollment. The links between participants and their identities will be kept on password protected excel sheets on that are also restricted to authorized study personnel. Data that is shared with external collaborators will be de-identified prior to sending and at least two members of the study team will review the data to confirm that no PHI is present.

All data transfers will be via secure cloud link such as Box.com.

Aim 2 Data: All survey data collected at both sites will be entered into a REDCap project housed at BUMC. Patients will be identified by unique identifier only, but a separate linking file (not in REDCap) will be kept at each site and not shared outside the institution. This linking file will be used to link survey data to the associated patient EHR data. When this data is shared with BMC for data analysis, only a HIPAA LDS will be transferred via secure cloud link such as Box.com.

The study monitor or other authorized representatives of the sponsor may inspect all documents and records required to be maintained by the investigator.

## 11.2 Source Documents

Source documents for this study will consist of electronic health record (EHR) data from each data collection site (Boston Medical Center and Northshore Medical Center). Data generated by the methods described in the protocol will NOT be recorded in the subjects' medical records and/or study progress notes. Data may be transcribed legibly on CRFs supplied for each subject or directly inputted into an electronic system or any combination thereof.

## 11.3 Case Report Forms

The study CRF will be the primary data collection instrument for the study. All data requested on the CRF will be recorded. All missing data will be explained. Questions will have a response option for "Subject chose not to answer" or "Not applicable".

CRF List:

| |
|---|
| Sociodemographics |
| ACP Knowledge |
| ACP Engagement |

| |
|---|
| Confidence in future care |
| Communication satisfaction |
| Decisional satisfaction |
| Decisional conflict |

## 11.4 Study Records Retention

Study records, both paper and electronic versions, will be retained, per BMC policy, for at least seven years after completion of the study.

## 11.5 Data Management

Boston Medical Center will serve as the study data repository. A dedicated REDCap database housed at BMC will be used to manage randomization and survey data entry across all sites. Data will be regularly checked for errors and completeness.

Survey Data

Survey data from each of the clinical sites will be transmitted via secure, institutionally approved methods to Boston Medical Center. Identifying information in REDCap will be limited to only what is necessary for study procedures, and these will only be accessed to conduct study activities (contact information for study interviews). According to standard REDCap protocols, all access will be subject to monitoring and reporting. Assurance of confidentiality of information will be made to all subjects. Data will be handled with the same confidentiality accorded to patients' medical records.

Natural Language Processing (NLP) Data

The RA and site-PI at each of our two sites, where data is being collected, will extract data every two months from the EHR and surveys. Each site will maintain and adhere to the process and procedures for the protection of human subjects and protected health information (PHI) for their covered entities. All data collected by the RAs will be stored in password protected servers. Participant identifiers will be kept in separate password protected files and a third linking file will be maintained. The linking file will also be password protected, access will be minimized, and a logging feature will be used to identify each user and instance of use. Only the minimum amount of PHI necessary will be collected from study participants. NLP data from each of the sites will be processed locally and then a HIPAA LDS of these data will be transferred via secure institutionally approved methods to Dana Farber Cancer Institute (DFCI) for data management and then to BMC to be merged with the rest of the study data repository. Data stored on the Dana Farber server will reside there only for the periods they are required to be there for study usage. Data will be securely removed from these servers on a per-item basis. Removed data will be securely transferred to BMC long-term servers for storage.

Specific procedures protecting subject confidentiality will be as follows:

1. Access to data files will be secured with a password-filing system (that logs entry) and is restricted to authorized staff only.
2. Necessary hard-copy records containing study data of any type will be kept in locked files.
3. Master lists linking subject information with ID number will be numbered consecutively and prepared before data collection (to ensure accurate accounting). These lists will be kept locked, in duplicate, with access only by the PIs and the other investigators at the site.
4. All project staff will sign an oath of confidentiality to ensure their understanding of the terms of confidentiality required. They will be trained in specific procedures to ensure confidentiality.
5. Sign-out procedures for all access to data files will be strictly enforced.
6. All reports and publications will preserve participants' anonymity.

# 12 Statistical Plan

## 12.1 Study Hypotheses

**<u>Aim 1</u>:** To test the effects of a PCE video intervention leveraging video decision aids on the quality of end-of-life care. We will conduct a SW-CRT to evaluate intervention effectiveness by comparing the following outcomes among 15,000 hospitalized patients: ACP documentation; preferences for resuscitation; palliative care consults; and, hospice use. ***Hypotheses****: A higher proportion of patients in the intervention phase (vs. control) will: (1) complete advance care plans* ***(primary outcome),*** *(2) have documented resuscitation preferences, (3) have palliative care consults, (4) enroll in hospice over the course of one year of follow-up, and (5) have documented health care proxies.*

**<u>Aim 2</u>:** To characterize caregiver-centered outcomes of patients with ADRD/Delirium, including: (1) knowledge, (2) confidence in future care, (3) communication satisfaction, (4) decisional satisfaction, and (5) decisional conflict in 600 caregivers of patients with ADRD/Delirium admitted to the hospital. ***Hypothesis****: Intervention phase caregivers of patients with ADRD*/Delirium *(vs. control) will have higher knowledge, confidence, communication satisfaction, decisional satisfaction, and lower decisional conflict.*

## 12.2 Sample Size Determination

**Statistical power and sample size:** All sample size estimates here assume a minimum of 80% power and a two-sided alpha of 0.05. We employ the method for the computation of sample size for cross-sectional stepped wedge studies comparing intervention to usual care in two-group statistical analyses. This method incorporates information on the number of steps used in the

stepped wedge/cluster randomized design, the number of subjects per time period, and the degree of clustering via the intraclass correlation coefficient (ICC) to compute the design effect, the factor by which the sample size found to provide sufficient statistical power for a meaningful intervention difference in outcome assuming independent data is multiplied. For the primary outcome of the documentation of ACP in the medical record, a sample size of 440 records per group in a chi-squared test for independent data will provide 80% power at a two-sided alpha of 0.05 to detect a difference in the proportion of subjects with notation of 35% in the intervention group compared to 25% in the usual care group, values consistent with prior research and expectation based on clinical data from the two health systems estimated from recent data. Based on our planned number of steps (7 with one uniformly applied usual care period across all hospital units), enrollment per study period, and a reasonable ICC of 0.01, the design effect is 2.72. Thus, we will need to obtain outcome data from the records of at least 2394 subjects overall (1197 per health system) to provide 80% power for our analysis of intervention effectiveness. We anticipate, however, that as many as 15,000 records will be available for analysis with respect to the documentation of ACP. Thus, our planned sample size for our primary records-based analysis on 15,000 records will therefore provide more than adequate power to test for differences in our primary outcome.

Data for Aim 1 is derived from the EHR and as is typical for trials that integrate new initiatives within the workflow of large institutions in a SW-CRT that does not involve consent. Indeed, we have been previously approved by multiple IRBs for such activities. Along with our exceedingly efficient NLP-assisted and human-confirmed software method for EHR data extraction, we can have a very large study sample with for this activity.

Please note: We anticipate the population under study in Aim 1 to exceed that required by a simple application of the power calculation presented above. However, this is warranted for eight reasons. First, the size of this observed population gives us the opportunity to examine intervention effects for less common outcomes. Second, this sample size will allow us to evaluate potential heterogeneity in treatment effects for subpopulations as small as 20% of the population. Third, this sample size provides an experimental context in which we will be able to recruit a population of 600 patients with ADRD/Delirium and their associate caregivers for surveying. Indeed, to sustain the activities of Aim 2, we need a large population to draw from, as many of the people under study for Aim 1 would not be eligible for participation in Aim 2. Fourth, the size of the population for Aim 1 also protects this trial from the potential that we will have significantly varying sizes of study clusters, a factor that is often neglected in sample size assessments for SW-CRTs.(145) Indeed, this is a likely phenomenon as hospital units vary significantly in their population of patients. Fifth, there is minimal risk to human subjects presented by the expanded sample size for Aim 1. Indeed, this educational intervention is being spread across the clinical units of our two hospitals in a pragmatic manner as part of the standard of care. The research activities of Aim 1 involve no direct burden to patients as there is no consent process and data for this activity will be derived from the EHR. The chief risk is the loss of confidentiality and robust protections are in place to protect patients from this potential risk. Sixth, we plan to extend this intervention as a new clinical initiative in our two health systems in a manner (time per cluster) that has been endorsed by leadership as a reasonable rate for dissemination (i.e., we are not adding more time). Seventh, we have devised an exceedingly efficient and accurate method for outcome assessment (i.e., we are not adding more

cost). Eighth, we will protect against inappropriate conclusions. We understand that treatment effect sizes will be more relevant than p-values and that clinical significance is the goal (not simply statistical significance).(146, 147) We have set an absolute increase of 10%, i.e., an increase of ACP documentation during the index hospitalization from 25% to 35%, as the benchmark for clinical significance. **In summary, the size of Aim 1 is needed to be able to do Aim 2b and we have taken appropriate measures to ensure that the research design for Aim 1 does not yield consequences for being overpowered.**

For the interview survey derived outcomes (knowledge, confidence in future care, communication satisfaction, decisional satisfaction, and decisional conflict) with approximately 600 subjects available across the 7 "clusters"/steps, the resulting design effect is 2.03 (again, assuming an ICC of 0.01). For this analysis sample size, the minimum effect size that can be detected for the uncertainty and knowledge scores separately with 90% power and alpha=0.05 would be 0.53 after applying the design effect. ***In sum, our anticipated sample sizes for both our primary and secondary aims will provide adequate statistical power to detect moderately sized and clinically important effects of the intervention and account for the cluster-randomized nature of our stepped wedge study design.***

## 12.3 Statistical Methods

**Statistical Analysis:** For the primary analyses of the primary and secondary outcomes, there will be no crossover of data for subjects from usual care to the intervention during the study; that is, subjects will only contribute data once during the course of the study, from their index hospitalization. Similarly, patients who move units during the course of their index hospitalization will be assigned to contribute intervention time data if they spend at least eight daytime weekday hours after being identified as meeting the inclusion criterion on a clinical unit where the intervention is being conducted. Accordingly, data being contributed by patients at each site during the pre-intervention period and data being contributed by patients after the initiation of the intervention will be kept separate for initial analyses. However, because we expect some patients to have multiple hospitalizations during different steps or to different units (i.e., crossover design), we will perform secondary analyses on all outcomes including data from the index hospitalization. This will include stratified sensitivity analyses of patients who contribute data (a) only to control period; (b) only to intervention period; or, (c) to both control and intervention periods.

Given the randomized nature of the stepped wedge design, we will report our results according to CONSORT guidelines. For the aims of the study that require patient/caregiver enrollment (Aim 2), we will record the number of people approached, screened, ineligible, and refusing participation. We will record subject attrition and note all adverse events. We will employ the intent-to-treat principle in our comparative analyses between the intervention and usual care groups. All hypothesis tests will employ a two-sided alpha level of 0.05. Given that the primary aim will be addressed by the analysis of data obtained from available patient records for the study period, we will examine the distributions of relevant variables focusing on the data relating to the documentation of ACP, the outcome of this aim. For the secondary aims of the study that will require enrollment of a caregiver sample for interview (Aim 2), we will examine the

distributions of the uncertainty and knowledge scale scores, the outcomes of interest between intervention and usual care subjects, as well as the distributional characteristics of all other salient study variables. We will generate descriptive statistics (means, standard deviations, quantiles for continuous variables; counts and percentages for categorical variables) and schematic plots (box-and-whisker, quantile-quantile plots). Given the nature of the cluster randomization that we will employ, we will utilize statistical analytic methods that take the correlated nature of the data into account as well as the influence of time to account for secular trends. In this study, we will examine both the health system and hospital unit as clustering variables, with the hospital unit as the primary clustering variable. We will compare the intervention and usual care groups on salient variables in order to assess balance in the distributions of these variables. Variables found to differ between the study groups will be further evaluated to assess their confounding effects of intervention vs. usual care differences on outcomes in multivariable analyses for correlated (clustered) data.

**Aim 1.** To test the combined effects of a COVID-19 ACP Educator-led, video-assisted palliative care intervention on rates of: ACP documentation; Medical orders for resuscitation preferences in the EHR; Palliative Care Consults; and, Hospice use. ***Hypothesis****: A higher proportion of patients in the intervention phase (vs. control) will: complete ACP documentation* ***(primary trial outcome),*** *have documented orders for resuscitation preferences, have palliative care consults, and enroll in hospice.*

*Primary outcome: ACP documentation.* In order to formally estimate and test differences in the proportion of patients with documentation of ACP between the intervention and usual care groups, we will employ logistic regression models for correlated binary outcome data. These models will either involve the use of robust variance methods to account for the clustering of these data by hospital site and/or health system via generalized estimating equations (GEE) or the inclusion of a random effects terms (in which case, the results will be interpreted as cluster-specific). Other potential modifiers of the effect of intervention, confounding variables, or covariates can be added to this model as fixed effects. Although we do not expect effect modification in the study data, we will examine the potential for such effects (interaction) through the use of stratified analyses and the inclusion of interaction terms with study group in our statistical models. Candidate effect modifiers will be specified a priori and will include age, gender, race/ethnicity, religion, and language. We will also examine and incorporate secular trend effects, i.e., the effect of time over the course of the study. Statistically significant interactions with the intervention will be retained and the nature of heterogeneous intervention effects will be estimated using the interaction model.

Based on our prior work in which we exhibited the fact that African-American and Hispanic patients are at particularly high risk for lower level of knowledge related to ACP, not discussing ACP with family, not having a health care proxy, and not having ACP documentation, we anticipate that this intervention may be particularly beneficial for African-American and Hispanic patients.(115, 131, 132) Accordingly, we will evaluate heterogeneous treatment effects by race and ethnicity and anticipate having adequate diversity in our study population to make such assessments. All data regarding Aim 1 will come from the EHR. Our institutions maintain excellent self-report information regarding race and ethnicity.

We will conduct analyses related to potential effect modification as a step in our model validation process and to identify relationships that can be examined more fully in future research. Should interactions not be found to be statistically significant, we will fit a main effects-only model and use it to formally evaluate confounding by applying a change-in-estimates approach, with a 10% change in estimates being an initial screening criterion. *Secondary outcomes*: Similar procedures will be undertaken to assess intervention effects for the other EHR derived outcomes (documented orders for resuscitation preferences, palliative care consults, hospice enrollment, and documented health care proxies).

For our primary analysis, we will consider our primary outcome (ACP documentation) and our secondary outcomes (resuscitation preferences, palliative care, hospice use, and health care proxies) only for the patient's index hospitalization. However, because we expect some patients to have multiple rehospitalizations during the same step and may also include intervention time (i.e., crossover design), we will perform secondary analyses on all of our primary and secondary outcomes for each patient reviewing <u>all</u> EHR records from the index hospitalization of the patient until their death (or through study period). We will also perform stratified sensitivity analyses of patients who contribute only to control period vs. patients who contribute only to intervention period vs. those that contribute to both control and intervention periods.

We will conduct the above analyses on all Aim 1 and Aim 2a patients for the study primary and secondary outcomes.

**Aim 2b.** To characterize detailed caregiver-centered outcomes, including knowledge, confidence in future care, communication and decisional satisfaction, and decisional certainty in a group of caregivers of patients with ADRD/Delirium admitted to the hospital. ***Hypothesis****: Caregivers in the intervention phase (vs. control) will have higher knowledge, confidence in future care, improved communication and decisional satisfaction, and less decisional conflict.* For Aim 2b, we will compare survey responses from intervention and control periods to take into account clustering within clinical unit and hospital. We will include calendar time and any imbalance from caregiver characteristics in the model to adjust for the potential confounding factors. We will account for clustering using methods as described above but will employ linear models for correlated data fitted via GEE or in mixed models.

**Missing data:** We will impute data when missing using multiple imputation techniques. This approach is one of the statistically principled methods noted in a recent *NEJM* editorial on the need for such approaches in the analysis of data from RCTs with missing values.(141) This approach assumes that data are missing either completely at random (MCAR) or at random (MAR) as a function of non-missing data on available variables in the dataset. We will implement this process using PROC MI in SAS. We will generate 20 imputed datasets and will conduct our intent-to-treat analyses per our analysis plan, saving results across datasets so they can be combined using PROC MIANALYZE in SAS. We will also consider the possibility that data are missing in a non-ignorable fashion. For example, should more or less symptomatic subjects be lost to follow-up as a result of treatment – and thus produce results that are biased in a manner not addressable by the above methods that assume MCAR or MAR data – we will randomly

impute data in sensitivity analyses under various alternative scenarios employing multiple imputation with the combination of analytic results noted above.

Reporting dropout and missing data. Whenever a participant in the caregiver interview sample drops out of the study, we will document the specific reason for dropout, who decided that the participant would drop out, and whether the dropout involved intervention participation, data collection, or both. If a participant withdraws from the intervention only, we will continue to collect data on all outcome measures. All participants included will be accounted for in a CONSORT diagram.

## 13 Ethics/Protection of Human Subjects

This study is to be conducted according to applicable U.S. federal regulations and institutional policies (which are based in federal regulations, guidance, and ICH Good Clinical Practice guidelines).

This protocol and any amendments will be submitted to the BMC IRB, for formal approval of the study conduct. The decision of the IRB concerning the conduct of the study will be made in writing to the investigator. A copy of the initial IRB approval letter will be provided to the sponsor before commencement of this study.

All subjects enrolled for Aim 2 (caregiver survey) will provide verbal informed consent by phone prior to answering any survey questions. Subjects will be provided with sufficient information and time to make an informed decision about their participation in this study. These subjects will be offered to have a copy of the consent form mailed to them (Email or US Mail) to keep for their records. The consent form will be submitted with the protocol for review and approval by the IRB. The consent of a subject, using the IRB-approved consent form, must be obtained before that subject is submitted to any study procedure. Consent will be documented as required by the IRB.

## Literature References

1. Berger NA, Savvides P, Koroukian SM, et al. Cancer in the elderly. *Transactions of the American Clinical and Climatological Association*. 2006;117:147-55; discussion 155-6.
2. Richardson S, Hirsch JS, Narasimhan M, et al. Presenting Characteristics, Comorbidities, and Outcomes Among 5700 Patients Hospitalized With COVID-19 in the New York City Area. *JAMA*. Apr 22 2020;doi:10.1001/jama.2020.6775
3. Yabroff KR, Lamont EB, Mariotto A, et al. Cost of care for elderly cancer patients in the United States. *Journal of the National Cancer Institute*. May 07 2008;100(9):630-41. doi:10.1093/jnci/djn103
4. Weissman GE, Crane-Droesch A, Chivers C, et al. Locally Informed Simulation to Predict Hospital Capacity Needs During the COVID-19 Pandemic. *Ann Intern Med*. Jul 7 2020;173(1):21-28. doi:10.7326/M20-1260
5. Dumanovsky T, Augustin R, Rogers M, Lettang K, Meier DE, Morrison RS. The Growth of Palliative Care in U.S. Hospitals: A Status Report. *Journal of palliative medicine*. Jan 2016;19(1):8-15. doi:10.1089/jpm.2015.0351
6. Dumanovsky T, Rogers M, Spragens LH, Morrison RS, Meier DE. Impact of Staffing on Access to Palliative Care in U.S. Hospitals. *Journal of palliative medicine*. Dec 2015;18(12):998-9. doi:10.1089/jpm.2015.0436
7. Hua M, Ma X, Morrison RS, Li G, Wunsch H. Association between the Availability of Hospital-based Palliative Care and Treatment Intensity for Critically Ill Patients. *Annals of the American Thoracic Society*. Sep 2018;15(9):1067-1074. doi:10.1513/AnnalsATS.201711-872OC
8. Rush B, Berger L, Anthony Celi L. Access to Palliative Care for Patients Undergoing Mechanical Ventilation With Idiopathic Pulmonary Fibrosis in the United States. *The American journal of hospice & palliative care*. Mar 2018;35(3):492-496. doi:10.1177/1049909117713990
9. Wong TW, Lang-Brown S, Romo RD, et al. Prognosis Communication in Late-Life Disability: A Mixed Methods Study. *J Am Geriatr Soc*. Nov 2017;65(11):2496-2501. doi:10.1111/jgs.15025
10. Spetz J, Dudley N, Trupin L, Rogers M, Meier DE, Dumanovsky T. Few Hospital Palliative Care Programs Meet National Staffing Recommendations. *Health affairs (Project Hope)*. Sep 1 2016;35(9):1690-7. doi:10.1377/hlthaff.2016.0113
11. O'Mahony S, Levine S, Baron A, et al. Palliative Workforce Development and a Regional Training Program. *The American journal of hospice & palliative care*. Jan 2018;35(1):138-143. doi:10.1177/1049909116685046
12. Pawlow P, Dahlin C, Doherty CL, Ersek M. The Hospice and Palliative Care Advanced Practice Registered Nurse Workforce: Results of a National Survey. *Journal of hospice and palliative nursing : JHPN : the official journal of the Hospice and Palliative Nurses Association*. Aug 2018;20(4):349-357. doi:10.1097/njh.0000000000000449
13. Dudley N, Chapman S, Spetz J. Community-Based Palliative Care Leader Perspectives on Staffing, Recruitment, and Training. *Journal of hospice and palliative nursing : JHPN : the official journal of the Hospice and Palliative Nurses Association*. Apr 2018;20(2):146-152. doi:10.1097/njh.0000000000000419
14. Szekendi MK, Vaughn J, Lal A, Ouchi K, Williams MV. The Prevalence of Inpatients at 33 U.S. Hospitals Appropriate for and Receiving Referral to Palliative Care. *Journal of palliative medicine*. Apr 2016;19(4):360-72. doi:10.1089/jpm.2015.0236
15. Patel RV, Kelley AS, Kamal AH. The Denominator: Evolving the Electronic Medical Record To Discover Who Needs Palliative Care. *Journal of palliative medicine*. Jan 2018;21(1):9-10. doi:10.1089/jpm.2017.0382
16. Fadul N, Elsayem AF, Bruera E. Integration of palliative care into COVID-19 pandemic planning. *BMJ Support Palliat Care*. Jun 11 2020;doi:10.1136/bmjspcare-2020-002364

17. Wendler D, Rid A. Systematic review: the effect on surrogates of making treatment decisions for others. *Ann Intern Med*. Mar 1 2011;154(5):336-46. doi:10.7326/0003-4819-154-5-201103010-00008

18. Shalowitz DI, Garrett-Mayer E, Wendler D. The accuracy of surrogate decision makers: a systematic review. *Arch Intern Med*. Mar 13 2006;166(5):493-7. doi:10.1001/archinte.166.5.493

19. Cohen G, Russo MJ, Campos JA, Allegri RF. Living with dementia: increased level of caregiver stress in times of COVID-19. *Int Psychogeriatr*. Jul 30 2020:1-11. doi:10.1017/S1041610220001593

20. Park CL, Russell BS, Fendrich M, Finkelstein-Fox L, Hutchison M, Becker J. Americans' COVID-19 Stress, Coping, and Adherence to CDC Guidelines. *J Gen Intern Med*. Aug 2020;35(8):2296-2303. doi:10.1007/s11606-020-05898-9

21. Kent EE, Ornstein KA, Dionne-Odom JN. The Family Caregiving Crisis Meets an Actual Pandemic. *J Pain Symptom Manage*. Jul 2020;60(1):e66-e69. doi:10.1016/j.jpainsymman.2020.04.006

22. Dying in America: improving quality and honoring individual preferences near the end of life. *Mil Med*. Apr 2015;180(4):365-7. doi:10.7205/MILMED-D-15-00005

23. Davison SN. Advance care planning in patients with chronic kidney disease. *Semin Dial*. Nov-Dec 2012;25(6):657-63. doi:10.1111/sdi.12039

24. Goff SL, Eneanya ND, Feinberg R, et al. Advance Care Planning: A Qualitative Study of Dialysis Patients and Families. *Clin J Am Soc Nephrol*. Feb 13 2015;doi:10.2215/cjn.07490714

25. Braun UK, McCullough LB, Beyth RJ, Wray NP, Kunik ME, Morgan RO. Racial and ethnic differences in the treatment of seriously ill patients: a comparison of African-American, Caucasian and Hispanic veterans. *J Natl Med Assoc*. Sep 2008;100(9):1041-51.

26. Cohen LM, Germain M, Poppel DM, Woods A, Kjellstrand CM. Dialysis discontinuation and palliative care. *Am J Kidney Dis*. Jul 2000;36(1):140-4. doi:10.1053/ajkd.2000.8286

27. Cohen LM, Germain MJ, Poppel DM. Practical considerations in dialysis withdrawal: "to have that option is a blessing". *Jama*. Apr 23-30 2003;289(16):2113-9. doi:10.1001/jama.289.16.2113

28. Cohen LM, Ruthazer R, Germain MJ. Increasing hospice services for elderly patients maintained with hemodialysis. *J Palliat Med*. Jul 2010;13(7):847-54. doi:10.1089/jpm.2009.0375

29. Gillick MR. Advance care planning. *N Engl J Med*. Jan 1 2004;350(1):7-8. doi:10.1056/NEJMp038202

30. Tulsky JA. Interventions to enhance communication among patients, providers, and families. *J Palliat Med*. 2005;8 Suppl 1:S95-102. doi:10.1089/jpm.2005.8.s-95

31. Tulsky JA. Improving quality of care for serious illness: findings and recommendations of the Institute of Medicine report on dying in America. *JAMA Intern Med*. May 2015;175(5):840-1. doi:10.1001/jamainternmed.2014.8425

32. Volandes AE, Kennedy WJ, Davis AD, Gillick MR, Paasche-Orlow MK. The new tools: What 21st century education can teach us. *Healthc (Amst)*. Dec 2013;1(3-4):79-81. doi:10.1016/j.hjdsi.2013.07.011

33. Sudore R, Le GM, McMahan R, Feuz M, Katen M, Barnes DE. The advance care planning PREPARE study among older Veterans with serious and chronic illness: study protocol for a randomized controlled trial. *Trials*. Dec 12 2015;16:570. doi:10.1186/s13063-015-1055-9

34. Sudore R, Le GM, McMahon R, Feuz M, Katen M, Barnes DE. The advance care planning PREPARE study among older Veterans with serious and chronic illness: study protocol for a randomized controlled trial. *Trials*. 2015;16(1):570. doi:10.1186/s13063-015-1055-9

35. Sudore RL, Barnes DE, Le GM, et al. Improving advance care planning for English-speaking and Spanish-speaking older adults: study protocol for the PREPARE randomised controlled trial. *BMJ Open*. Jul 11 2016;6(7):e011705. doi:10.1136/bmjopen-2016-011705

36. Sudore RL, Boscardin J, Feuz MA, McMahan RD, Katen MT, Barnes DE. Effect of the PREPARE Website vs an Easy-to-Read Advance Directive on Advance Care Planning Documentation and Engagement Among Veterans: A Randomized Clinical Trial. *JAMA Intern Med*. Aug 1 2017;177(8):1102-1109. doi:10.1001/jamainternmed.2017.1607

37. Sudore RL, Knight SJ, McMahan RD, et al. A novel website to prepare diverse older adults for decision making and advance care planning: a pilot study. *J Pain Symptom Manage*. Apr 2014;47(4):674-86. doi:10.1016/j.jpainsymman.2013.05.023

38. Quintiliani LM MJ, Buitron de la Vega P, Waite K, Armstrong SE, Henault L, Volandes AE, Paasche-Orlow M. Feasibility and patient perceptions of video declarations regarding end-of-life decisions by hospitalized patients. . *J Palliat Med*. 2017;Accepted

39. Givens JL, Kiely DK, Carey K, Mitchell SL. Healthcare proxies of nursing home residents with advanced dementia: decisions they confront and their satisfaction with decision-making. *Journal of the American Geriatrics Society*. Jul 2009;57(7):1149-55. doi:10.1111/j.1532-5415.2009.02304.x

40. Teno JM, Clarridge BR, Casey V, et al. Family perspectives on end-of-life care at the last place of care. *JAMA : the journal of the American Medical Association*. Jan 7 2004;291(1):88-93. doi:10.1001/jama.291.1.88

41. Mack JW, Paulk ME, Viswanath K, Prigerson HG. Racial disparities in the outcomes of communication on medical care received near death. *Archives of internal medicine*. Sep 27 2010;170(17):1533-40. doi:10.1001/archinternmed.2010.322

42. Kelley AS, Ettner SL, Morrison RS, Du Q, Wenger NS, Sarkisian CA. Determinants of medical expenditures in the last 6 months of life. *Annals of internal medicine*. Feb 15 2011;154(4):235-42. doi:10.7326/0003-4819-154-4-201102150-00004

43. Teno JM, Gozalo PL, Bynum JP, et al. Change in end-of-life care for Medicare beneficiaries: site of death, place of care, and health care transitions in 2000, 2005, and 2009. *JAMA : the journal of the American Medical Association*. Feb 6 2013;309(5):470-7. doi:10.1001/jama.2012.207624

44. A controlled trial to improve care for seriously ill hospitalized patients. The study to understand prognoses and preferences for outcomes and risks of treatments (SUPPORT). The SUPPORT Principal Investigators. *JAMA : the journal of the American Medical Association*. Nov 22-29 1995;274(20):1591-8.

45. Smedira NG, Evans BH, Grais LS, et al. Withholding and withdrawal of life support from the critically ill. *The New England journal of medicine*. Feb 01 1990;322(5):309-15. doi:10.1056/nejm199002013220506

46. Wright AA, Zhang B, Ray A, et al. Associations between end-of-life discussions, patient mental health, medical care near death, and caregiver bereavement adjustment. *Jama*. Oct 8 2008;300(14):1665-73. doi:10.1001/jama.300.14.1665

47. Mitchell SL, Teno JM, Intrator O, Feng Z, Mor V. Decisions to forgo hospitalization in advanced dementia: a nationwide study. *Journal of the American Geriatrics Society*. Mar 2007;55(3):432-8. doi:10.1111/j.1532-5415.2007.01086.x

48. Mack JW, Weeks JC, Wright AA, Block SD, Prigerson HG. End-of-life discussions, goal attainment, and distress at the end of life: predictors and outcomes of receipt of care consistent with preferences. *Journal of clinical oncology : official journal of the American Society of Clinical Oncology*. Mar 1 2010;28(7):1203-8. doi:10.1200/jco.2009.25.4672

49. Detering KM, Hancock AD, Reade MC, Silvester W. The impact of advance care planning on end of life care in elderly patients: randomised controlled trial. *BMJ (Clinical research ed)*. 2010;340:c1345. doi:10.1136/bmj.c1345

50. Leung JM, Udris EM, Uman J, Au DH. The effect of end-of-life discussions on perceived quality of care and health status among patients with COPD. *Chest*. Jul 2012;142(1):128-33. doi:10.1378/chest.11-2222

51. Abel J, Pring A, Rich A, Malik T, Verne J. The impact of advance care planning of place of death, a hospice retrospective cohort study. *BMJ supportive & palliative care*. Jun 2013;3(2):168-73. doi:10.1136/bmjspcare-2012-000327

52. Silveira MJ, Kim SY, Langa KM. Advance directives and outcomes of surrogate decision making before death. *The New England journal of medicine*. Apr 1 2010;362(13):1211-8. doi:10.1056/NEJMsa0907901

53. Austin CA, Mohottige D, Sudore RL, Smith AK, Hanson LC. Tools to Promote Shared Decision Making in Serious Illness: A Systematic Review. *JAMA Intern Med*. Jul 2015;175(7):1213-21. doi:10.1001/jamainternmed.2015.1679

54. Volandes AE, Mitchell SL, Gillick MR, Chang Y, Paasche-Orlow MK. Using video images to improve the accuracy of surrogate decision-making: a randomized controlled trial. *J Am Med Dir Assoc*. Oct 2009;10(8):575-80. doi:10.1016/j.jamda.2009.05.006

55. Volandes AE, Paasche-Orlow M, Gillick MR, et al. Health literacy not race predicts end-of-life care preferences. *Journal of palliative medicine*. Jun 2008;11(5):754-62. doi:10.1089/jpm.2007.0224

56. Volandes AE, Paasche-Orlow MK, Barry MJ, et al. Video decision support tool for advance care planning in dementia: randomised controlled trial. *BMJ*. May 28 2009;338:b2159. doi:10.1136/bmj.b2159

57. Moss AH. Revised dialysis clinical practice guideline promotes more informed decision-making. *Clinical journal of the American Society of Nephrology : CJASN*. Dec 2010;5(12):2380-3. doi:10.2215/cjn.07170810

58. O'Hare AM, Song MK, Kurella Tamura M, Moss AH. Research Priorities for Palliative Care for Older Adults with Advanced Chronic Kidney Disease. *Journal of palliative medicine*. May 2017;20(5):453-460. doi:10.1089/jpm.2016.0571

59. Aitken PV, Jr. Incorporating advance care planning into family practice [see comment]. *Am Fam Physician*. Feb 01 1999;59(3):605-14, 617-20.

60. Emanuel LL, Barry MJ, Stoeckle JD, Ettelson LM, Emanuel EJ. Advance directives for medical care--a case for greater use. *N Engl J Med*. Mar 28 1991;324(13):889-95. doi:10.1056/NEJM199103283241305

61. Sudore RL, Fried TR. Redefining the "planning" in advance care planning: preparing for end-of-life decision making. *Ann Intern Med*. Aug 17 2010;153(4):256-61. doi:10.7326/0003-4819-153-4-201008170-00008

62. Tulsky JA, Beach MC, Butow PN, et al. A Research Agenda for Communication Between Health Care Professionals and Patients Living With Serious Illness. *JAMA Intern Med*. Jul 03 2017;doi:10.1001/jamainternmed.2017.2005

63. El-Jawahri A, Podgurski LM, Eichler AF, et al. Use of video to facilitate end-of-life discussions with patients with cancer: a randomized controlled trial. *Journal of clinical oncology : official journal of the American Society of Clinical Oncology*. Jan 10 2010;28(2):305-10. doi:10.1200/JCO.2009.24.7502

64. El-Jawahri A, Mitchell SL, Paasche-Orlow MK, et al. A Randomized Controlled Trial of a CPR and Intubation Video Decision Support Tool for Hospitalized Patients. *Journal of general internal medicine*. Aug 2015;30(8):1071-80. doi:10.1007/s11606-015-3200-2

65. Volandes AE, Paasche-Orlow MK, Mitchell SL, et al. Randomized controlled trial of a video decision support tool for cardiopulmonary resuscitation decision making in advanced cancer. *Journal of clinical oncology : official journal of the American Society of Clinical Oncology*. Jan 20 2013;31(3):380-6. doi:10.1200/JCO.2012.43.9570

66. Volandes AE, Ariza M, Abbo ED, Paasche-Orlow M. Overcoming educational barriers for advance care planning in Latinos with video images. *Journal of palliative medicine*. Jun 2008;11(5):700-6. doi:10.1089/jpm.2007.0172

67. Javier AD, Figueroa R, Siew ED, et al. Reliability and Utility of the Surprise Question in CKD Stages 4 to 5. *Am J Kidney Dis*. Jul 2017;70(1):93-101. doi:10.1053/j.ajkd.2016.11.025

68. Pfeiffer E. A short portable mental status questionnaire for the assessment of organic brain deficit in elderly patients. *J Am Geriatr Soc*. Oct 1975;23(10):433-41.

69. Moss AH. A new clinical practice guideline on initiation and withdrawal of dialysis that makes explicit the role of palliative medicine. *J Palliat Med*. Fall 2000;3(3):253-60. doi:10.1089/jpm.2000.3.253

70. Cameron AC, Trivedi PK. *Microeconometrics: Methods and Applications*. Cambridge University Press; 2005.

71. Hui D, Bruera E. Integrating palliative care into the trajectory of cancer care. *Nature reviews Clinical oncology*. Mar 2016;13(3):159-71. doi:10.1038/nrclinonc.2015.201

72. Creswell JW, Creswell JW, Elsie de Renzo Orlando Fund. *Qualitative inquiry and research design : choosing among five approaches*. 3rd ed. SAGE Publications; 2013:xxi, 448 p.

**Summary of VIDEO-PCE Trial Protocol Changes**

This supplement details all the amendments that were submitted throughout the course of the trial, including amendments to the caregiver survey activity which was conducted throughout the course of the trial but whose results are not presented in this manuscript.

**PROTOCOL REVISION (07/21/2021)**

Summary: This amendment revised the consent form for the caregiver survey activity and finalized the caregiver survey consent form for North Shore University Hospital. Additionally, Dana-Farber Cancer Institute was removed from sections 9.3 and 9.4 of the protocol as it was determined that Dana-Farber Cancer Institute was not engaged in research.

Rationale for Changes: These changes were submitted so that the protocol and consent forms best reflected trial activities.

**PROTOCOL REVISION (09/27/2021)**

Summary: To facilitate remote and continued access to the video decision aids, the trial protocol was amended to allow inpatient care team members to provide a handout to participants on the intervention units. This handout provided instructions for accessing the video decision aids after their discharge.

Additionally, this amendment revised the recruitment letter that was mailed to eligible caregivers from whom we had a mailing address. This revised recruitment letter indicated that a study staff member would call them within one week of the discharge date to ask if they would like to participate. For eligible caregivers for whom there was no mailing address, we called them asking if we can send a letter describing the study.

Rationale for Changes: Participants may want to review the video decision aids again at home or in the future with another clinician. Through providing a handout with instructions about how to access the video decisions aids after discharge, participants are empowered to return to the video as they wish.

Per the study protocol, the caregiver survey needs to be completed within one week of the hospital discharge. The original letter does not allow for that short time period as it indicated that caregivers would be called in two weeks if they did not return the opt out postcard. This amendment clarified that discrepancy.

**PROTOCOL REVISION (12/02/2021)**

Summary: To approve a Spanish language version of the caregiver survey. The survey was translated by Datagain and a certificate of accuracy was submitted with the amendment.

Rationale for Changes: To expand the reach of the caregiver survey activity to Spanish speaking caregivers.

**PROTOCOL REVISION (02/04/2022)**

Summary: Additional units were added to recruit caregivers form for the purpose of completing the caregiver survey activity. The inpatient diagnosis of delirium was added to the list of eligible diagnoses for the caregiver survey activity.

Rationale for Changes: To increase recruitment for caregiver surveys, additional non-study units were added to recruit control participants and the list of eligible diagnoses was expanded. Three additional

non-study units were added at Boston Medical Center and six were added at North Shore University Hospital.

## PROTOCOL REVISION (06/02/2022)

Summary: This amendment increased the worldwide study sample size from 9,600 to 15,600.

Rationale for Changes: The sample size in the original application was an estimate based on retroactive data reports of potentially eligible participants. In June 2022, when nearly a year of study data was collected, we realized that the actual number of eligible participants was higher than we originally projected.

## PROTOCOL REVISION (10/07/2022)

Summary: Through this amendment, two new study activities were added: 1) an update to the HIPPA section of the protocol to include an NLP analysis of inpatients notes of patients whose caregiver completed a survey; 2) a manual chart review activity for the patients of all caregivers enrolled in the caregiver survey.

Rationale for Changes: The expansion of our NLP analysis and the addition of a manual chart review activity allowed us to better study outcomes for patients whose caregiver completed a survey.
