# Supplementary material for: Video Intervention and Goals-of-Care Documentation in Hospitalized Older Adults: The VIDEO-PCE Randomized Clinical Trial
Source: JAMA Netw Open. 2023 Sep 11;6(9):e2332556. doi: 10.1001/jamanetworkopen.2023.32556 (PMC10495866; doi:10.1001/jamanetworkopen.2023.32556)
Supplement: Supplement 2. — eFigure. Protocol for PCE Patient Interaction eTable 1. VIDEO-PCE Trial Intervention Timeline eTable 2. Natural Language Processing Keyword Library eTable 3. Primary Discharge Diagnosis of Eligible Patients eTable 4. List of ICD-10 Diagnosis Codes Used to Identify ADRD Patients [file jamanetwopen-e2332556-s002.pdf]

Supplemental Online Content

Volandes AE, Zupanc SN, Lakin JR, et al. Effect of a video intervention on goals-of-care documentation in hospitalized older adults: the VIDEO-PCE randomized clinical trial. *JAMA Netw Open*. 2023;6(9):e2332556. doi:10.1001/jamanetworkopen.2023.32556

eFigure. Protocol for PCE Patient Interaction
eTable 1. VIDEO-PCE Trial Intervention Timeline
eTable 2. Natural Language Processing Keyword Library
eTable 3. Primary Discharge Diagnosis of Eligible Patients
eTable 4. List of *ICD-10* Diagnosis Codes Used to Identify ADRD Patients

This supplemental material has been provided by the authors to give readers additional information about their work.

© 2023 Volandes AE et al. *JAMA Network Open*.

eFigure. Protocol for PCE Patient Interaction

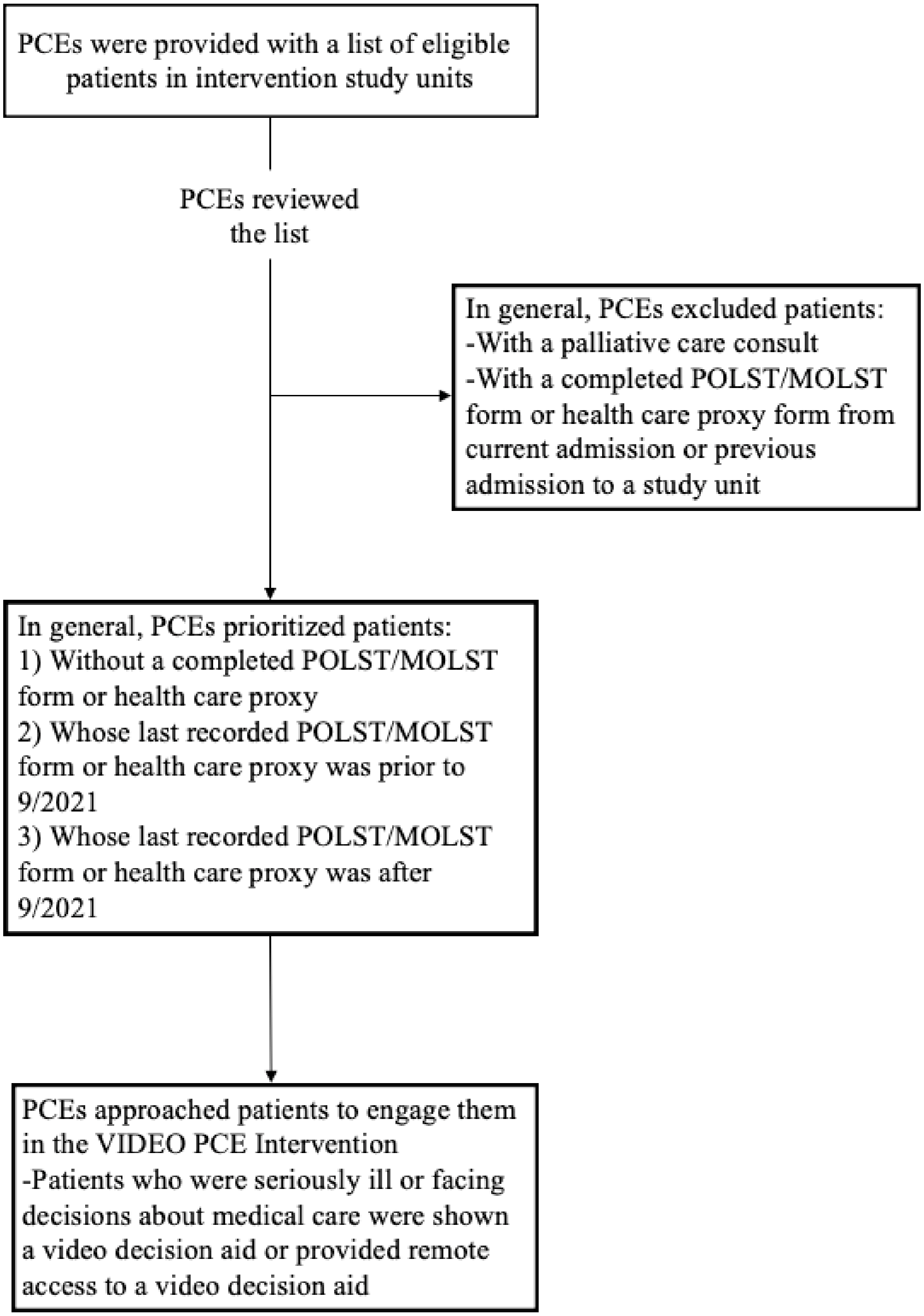

© 2023 Volandes AE et al. *JAMA Network Open*.

eTable 1. VIDEO-PCE Trial Intervention Timeline

Cluster	7/1/21 - 8/31/21	9/1/21 - 10/31/21	11/1/21 - 12/31/21	1/1/22 - 2/28/22	3/1/22 - 4/30/22	5/1/22- 6/30/22	7/1/22 - 8/31/22	9/1/22 - 10/31/22
1								
2								
3								
4								
5								
6								
7								

Intervention
Usual Care

© 2023 Volandes AE et al. *JAMA Network Open*.

eTable 2. Natural Language Processing Keyword Library

<table>
<tr><th>Annotation Domain</th><th>Definition</th><th>BMC Keywords</th><th>NHS Keywords</th></tr>
<tr><td>Goals Conversation</td><td>Conversations with patients or family members about the patient’s goals,
values, or priorities for treatment and outcomes. Includes statements that
conversation occurred as well as listing specific goals.

OR

Advance care planning was discussed, reviewed, or completed.</td><td>GOC, goals of care, goals for care, family meeting, family discussion, patient goals,
patient values, quality of life, prognostic discussions, illness understanding, serious illness conversation, serious illness discussion, advance care planning, ACP, end of life, what matters most, poor prognosis, limited prognosis, prognosis, prognostic, terminal, dying, die, death, incurable, not curable, not curative, non curable, non-curable, non curative, non-curative, no cure, isn’t a cure, is no cure, not reversible, non-reversible, treatments are palliative, treatment is palliative, palliative treatment, palliation, extend life, extending life, life-extending, life extending, lengthening life, lengthen life, life-lengthening, life lengthening, life limiting, life-limiting, does not wish to know, does not want to know, hours to days, days to weeks, weeks to months, months to years, prognostic understanding, month left, months left, years left, year left, weeks left, week left, unfortunate, regrettably, I am afraid, frank discussion, frank conversation, honest discussion, honest conversation, difficult conversation, difficult discussion, out of options, no remaining options, no more therapy, no further treatment, no further therapy, supportive care, comfort care, comfort approach, CMO, comfort directed care, prioritize comfort, end of life care, comfort measures, limiting invasive procedures, limit invasive procedures, what matters most, discussed, molst, polst, advanced care, advanced directive, advanced directives</td><td>GOC, goals of care, goals for care, family meeting, family discussion, patient goals, patient values, quality of life, prognostic discussion, illness understanding, serious illness conversation, serious illness discussion, advance care planning, (?<!I have reviewed the)(?<!documented by the)(?<!d\\/?w)ACP|(?<!documented by the)(?<!d\\/?w)ACP(?! note), end of life, what matters most, poor prognosis, limited prognosis, prognosis, prognostic, terminal, dying, die, incurable, not curable, not curative, non curable, non-curable, non curative, non-curative, no cure, isn’t a cure, is no cure, not reversible, non-reversible, treatments are palliative, treatment is palliative, palliative treatment, palliation, extend life, extending life, life-extending, life extending, lengthening life, lengthen life, life-lengthening, life lengthening, life limiting, life-limiting, does not wish to know, does not want to know, hours to days, days to weeks, weeks to months, months to years, prognostic understanding, month left, months left, years left, year left, weeks left, week left, unfortunate, regrettably, I am afraid, frank discussion, frank conversation, honest discussion, honest conversation, difficult conversation, difficult discussion, out of options, no remaining options, no more therapy, no further treatment, no further therapy, supportive care, comfort care, comfort approach, CMO, comfort directed care, prioritize comfort, end of life care, (?<!Treatment:)comfort measures(?! provided), limiting invasive procedures, limit invasive procedures, what matters most, molst, polst, advanced care, advanced directive, advanced directives, QOL</td></tr>
<tr><td>Limitations on
Life-Sustaining Treatments</td><td>Conversations with patients or family members about preferences for
limitations to cardiopulmonary resuscitation and intubation.</td><td>Full code, FC, full intubation, full recusitation, Intubation, resuscitation, CPR, no intubation, no resuscitation, no CPR, declines CPR, do not intubate, do not resuscitate, DNR/DNI, DNR, DNI, declines intubation, declines cardiopulmonary resuscitation, no chest compressions, no compressions, no defibrillation, no mechanical intubation, refuses intubation, refuses CPR, code status discussion, discussed code status, life support, DNAR, do not attempt resuscitation, vent, code status:</td><td>Full code, FC, full intubation, full recusitation, (?<!AMS;)intubation, (?<!IVF)resuscitation, CPR, no intubation, no resuscitation, no CPR, declines CPR, do not intubate, do not resuscitate, DNR/DNI, DNR, DNI, declines intubation, declines cardiopulmonary resuscitation, no chest compressions, no compressions, no defibrillation, no mechanical intubation, refuses intubation, refuses CPR, code status discussion, discussed code status, life support, DNAR, do not attempt resuscitation, code status:</td></tr>
<tr><td>Palliative Care</td><td>Mention of a visit with a specialty palliative care clinician, mention of
specialist palliative care discussion, or patient preferences regarding seeing a
palliative care clinician.</td><td>Palliative care, palliative medicine, pall care, pal care, pallcare, palcare, PC</td><td>Palliative care, palliative medicine, pall care, pal care, pallcare, palcare, PC</td></tr>
<tr><td>Time-Limited Trial</td><td>Conversations with patients or family members about the use of a treatment
or procedure for a set amount of time.</td><td>time-limited trial, time limited trial, limited trial, TLT</td><td>time-limited trial, time limited trial, limited trial, TLT</td></tr>
<tr><td>Hospice</td><td>Statements mentioning a discussion of hospice, prior enrollment in hospice,
patient preferences regarding hospice, or assessments of hospice eligibility.</td><td>Hospice, bridge to hospice, home hospice, inpatient hospice, hospice house, hospice at home</td><td>Hospice(?! patient\\s*\\|\\s*No\\s*\\|)(?! \\[\\s*\\]), bridge to hospice, home hospice, inpatient hospice, hospice house, hospice at home</td></tr>
</table>

© 2023 Volandes AE et al. *JAMA Network Open*.

eTable 3. Primary Discharge Diagnosis of Eligible Patients

	No. (%)		
	**Usual Care (n = 4779)**	**Intervention (n = 6023)**	**Overall (n = 10,802)**
**Primary Discharge Diagnosis**			
Certain Infectious and Parasitic Diseases	349 (7.3%)	372 (6.2%)	721 (6.7%)
Neoplasms	129 (2.7%)	215 (3.6%)	344 (3.2%)
Diseases of the Blood and Blood-Forming Organs and Certain Disorders Involving the Immune Mechanism	116 (2.4%)	142 (2.4%)	258 (2.4%)
Endocrine, Nutritional and Metabolic Diseases	199 (4.2%)	221 (3.7%)	420 (3.9%)
Mental, Behavioral and Neurodevelopmental Disorders	36 (0.8%)	64 (1.1%)	100 (0.9%)
Diseases of the Nervous System	116 (2.4%)	158 (2.6%)	274 (2.5%)
Diseases of the Eye and Adnexa	7 (0.1%)	12 (0.2%)	19 (0.2%)
Diseases of the Ear and Mastoid Process	3 (0.1%)	6 (0.1%)	9 (0.1%)
Diseases of the Circulatory System	1234 (25.8%)	1287 (21.4%)	2521 (23.3%)
Diseases of the Respiratory System	354 (7.4%)	475 (7.9%)	829 (7.7%)
Diseases of the Digestive System	384 (8.0%)	501 (8.3%)	885 (8.2%)
Diseases of the Skin and Subcutaneous Tissue	91 (1.9%)	121 (2.0%)	212 (2.0%)
Diseases of the Musculoskeletal System and Connective Tissue	138 (2.9%)	222 (3.7%)	360 (3.3%)
Diseases of the Genitourinary System	315 (6.6%)	415 (6.9%)	730 (6.8%)
Congenital Malformations, Deformations, and Chromosomal Abnormalities	1 (0.0%)	2 (0.0%)	3 (0.0%)
Symptoms, Signs, and Abnormal Clinical and Laboratory Findings, Not Elsewhere Classified	774 (16.2%)	1105 (18.3%)	1879 (17.4%)
Injury, Poisoning, and Certain Other Consequences of External Causes	281 (5.9%)	324 (5.4%)	605 (5.6%)
Codes for special purposes	112 (2.3%)	226 (3.8%)	338 (3.1%)
External Causes of Morbidity	46 (1.0%)	65 (1.1%)	111 (1.0%)
Factors Influencing Health Status and Contact with Health Services	69 (1.4%)	72 (1.2%)	141 (1.3%)
Missing/Not Available	25 (0.5%)	18 (0.3%)	43 (0.4%)

© 2023 Volandes AE et al. *JAMA Network Open*.

eTable 4. List of *ICD-10* Diagnosis Codes Used to Identify ADRD Patients

Code	Code Description
F01.50	Vascular dementia without behavioral disturbance
F01.51	Vascular dementia with behavioral disturbance
F02.80	Dementia in other diseases classified elsewhere without behavioral disturbance
F02.81	Dementia in other diseases classified elsewhere with behavioral disturbance
F03.90	Unspecified dementia without behavioral disturbance
F03.91	Unspecified dementia with behavioral disturbance
G30.0	Alzheimer's disease with early-onset
G30.1	Alzheimer's disease with late-onset
G30.8	Other Alzheimer's disease
G30.9	Alzheimer's disease, unspecified
G31.01	Pick's disease
G31.09	Other frontotemporal dementia
G31.1	Senile degeneration of brain, not elsewhere classified
G31.2	Degeneration of nervous system due to alcohol
G31.81	Alpers disease
G31.82	Leigh's disease
G31.83	Dementia with Lewy bodies
G31.84	Mild cognitive impairment, so stated
G31.85	Corticobasal degeneration
G31.89	Other specified degenerative diseases of nervous system
G31.9	Degenerative disease of nervous system, unspecified
G32.0	Subacute combined degeneration of spinal cord in diseases classified elsewhere
G32.81	Cerebellar ataxia in diseases classified elsewhere
G32.89	Other specified degenerative disorders of nervous system in diseases classified elsewhere
R41.3	Memory changes
I69.311	Memory deficit after cerebral infarction
I69.911	Memory deficit following unspecified cerebrovascular disease
I69.111	Memory deficit following nontraumatic intracerebral hemorrhage
I69.011	Memory deficit following nontraumatic subarachnoid hemorrhage
I69.811	Memory deficit following other cerebrovascular disease
I69.211	Memory deficit following other nontraumatic intracranial hemorrhage
F09	Memory disorder due to organic damage
R41.0	Disorientation, unspecified
F05	Delirium due to known physiological condition

© 2023 Volandes AE et al. *JAMA Network Open*.
