# Supplementary material for: Video Intervention and Goals-of-Care Documentation in Hospitalized Older Adults: The VIDEO-PCE Randomized Clinical Trial
Source: JAMA Netw Open. 2023 Sep 11;6(9):e2332556. doi: 10.1001/jamanetworkopen.2023.32556 (PMC10495866; doi:10.1001/jamanetworkopen.2023.32556)
Supplement: Supplement 3. — Data Sharing Statement [file jamanetwopen-e2332556-s003.pdf]

Data Sharing Statement

Volandes. Effect of a Video Intervention on Goals-of-Care Documentation in Hospitalized Older Adults. *JAMA Netw Open*. Published September 11, 2023.
doi:10.1001/jamanetworkopen.2023.32556

Data
Data available: No

Additional Information
Explanation for why data not available: The data will not be shared because we do not have permission from the involved healthcare systems to share the data.
